# Targeting mitochondrial one-carbon enzyme MTHFD2 together with pemetrexed confers therapeutic advantages in lung adenocarcinoma

**DOI:** 10.1038/s41420-022-01098-y

**Published:** 2022-07-05

**Authors:** Juanfen Mo, Zhenzhen Gao, Li Zheng, Miaolong Yan, Min Xue, Jianqiu Xu, Yi Bao, Jiayuan Wu

**Affiliations:** 1 The Key Laboratory, The Second Affiliated Hospital of Jiaxing University, Jiaxing, 314000 Zhejiang China; 2 Department of Oncology, The Second Affiliated Hospital of Jiaxing University, Jiaxing, 314000 Zhejiang China; 3 Department of Pathology, The Second Affiliated Hospital of Jiaxing University, Jiaxing, 314000 Zhejiang China

**Keywords:** Lung cancer, Cell growth

## Abstract

Metabolic remodeling is the fundamental molecular feature of malignant tumors. Cancer cells require sufficient energy supplies supporting their high proliferative rate. MTHFD2, a mitochondrial one-carbon metabolic enzyme, is dysregulated in several malignancies and may serve as a promising therapeutic candidate in cancer treatment. Here, our data confirmed that MTHFD2 gene and protein was upregulated in the cancerous tissues of LUAD patients and was correlated with a poor survival in LUAD. MTHFD2 was involved in lung cancer cell proliferation, migration, and apoptosis by mediating its downstream molecules, such as DNA helicases (MCM4 and MCM7), as well as ZEB1, Vimentin and SNAI1, which contributed to tumor cell growth and epithelial-to-mesenchymal transition (EMT) process. Moreover, we identified that *miRNA-99a-3p* appeared to be an upstream mediator directly regulating MTHFD2 and MCM4 expression. Moreover, specific inhibition of MTHFD2 functions by siRNA or a chemical compound, improved anti-tumor sensitivities induced by pemetrexed in LUAD. Taken together, our study revealed the underlying molecular mechanisms of MTHFD2 in regulating cell proliferation and identified a novel therapeutic strategy improving the treatment efficacies in LUAD.

## Introduction

Lung cancer is still the most commonly diagnosed malignancy in worldwide [1]. Lung adenocarcinoma (LUAD) is the predominant histological subtype, which exhibits a high rate of recurrence, distant metastasis, and cancer-related death [2, 3]. Although substantial efforts have been made in the treatment of lung cancer, including surgical resection, chemotherapy, and immunotherapy, the 5-year survival rate is still <20% [4, 5]. Therefore, it is necessary to make efforts exploring more effective therapeutic strategies in LUAD.

Pemetrexed is the first-line chemotherapeutic drugs and frequently used together with cisplatin in the treatment of LUAD [6]. Pemetrexed is recognized as a folate antagonist targeting core enzymes required for synthesizing DNA precursors and plays anti-tumor activates in patients with LUAD [7]. The response rate of pemetrexed/cisplatin combination is around 41.3% in patients with non-small-cell lung cancer (NSCLC) [8]. In addition, decreased response to pemetrexed will be observed after a period of drug administration due to the fact that LUAD developed drug resistance. Therefore, it is valuable to investigate new agents which combined with pemetrexed are able to improve anti-tumor sensitivities in the treatment of LUAD.

Accumulating evidences suggest that the metabolic reprogramming is the essential molecular mechanism intrinsically linked to high speed proliferation of cancer cells [9–11]. One-carbon (1-C) unit is required for nucleotide synthesis and methylation reactions, that implicated in the proliferation rate across a variety of cancer cells [12–14]. MTHFD2 is integral to mitochondrial one-carbon metabolism via catalyzing the NAD^+^-dependent CH_2_-THF dehydrogenase and CH^+^-THF cyclohydrolase reactions within the mitochondria [15]. It directly catalyzes the transformation of methylenetetrahydrofolate and purine synthesis and provides sufficient precursors required for cell proliferation [16, 17]. Overexpression of MTHFD2 is reported in several malignancies and associated with tumor invasion, metastasis and poor prognosis, for instance, the increased expression of MTHFD2 is correlated with tumor growth in renal cell carcinoma, hepatocellular carcinoma, and lung cancer [18–20]. Unregulated MTHFD2 promotes malignant characteristics of cancer cells, but the underlying mechanisms and how it is regulated are still largely unknown.

In this study, we characterized the gene and protein expression of MTHFD2 in tumor tissues, and investigated the correlation between its transcriptional levels and clinical prognosis in patients with LUAD using TCGA database. Our study revealed the underlying molecular mechanisms of MTHFD2 regulating proliferation and identified a novel therapeutic strategy improving the treatment efficacies in LUAD.

## Materials and methods

### Lung tumor tissue samples

A total of 20 paired cancerous tissues and paracancerous tissues from LUAD patients were collected from the Second Affiliated Hospital of Jiaxing University. Each sample was histologically confirmed by experienced pathologists and diagnosed as LUAD before the study. Written informed consents were acquired from all patients. Each procedure in the present study was conducted according to the Declaration of Helsinki and approved by the ethic committee of the Second Affiliated Hospital of Jiaxing University (JXEY-2021JX156). Sample size was based on preliminary study or our previously published work.

### Cell culture and chemical compounds

Human lung adenocarcinoma A549, H1299, PC9 and human embryonic kidney 293 T cells lines were purchased from The Cell Bank of Type Culture Collection of Chinese Academy of Sciences (Shanghai, China). All the cell lines were maintained in DMEM (Solarbio, Shanghai, China) containing 10 % FBS (Hycolne, Logan, UT, USA) and penicillin/streptomycin (100 μg/mL) at 37 °C with 5% CO_2_.

A specific MTHFD2 inhibitor DS18561882 (HY-130251) and chemo-drug pemetrexed (HY-10820) were purchased from MedChemExpress (MCE, NJ, USA). To determine the half maximal inhibitory concentration (IC50) value, cells (3000 cells/well) were seeded into 96-well plates. 24 h later, cells were treated with increased concentrations of pemetrexed or DS18561882 (0, 0.039, 0.078, 0.156, 0.3125, 0.625, 1.25, 2.5, 5, 25, 50 µM) for 5 days. The IC50 value was calculated by MTT assay (absorbance wave length at 570 nm) and defined as the concentration of 50% cell growth inhibition compared with the untreated control cells.

### Small RNA interference

Small interfering RNAs (siRNAs) sequences targeting human *MTHFD2* were synthesized by GenePharma (Shanghai, China). Three different *MTHFD2* siRNA duplexes were tested, and *si-MTHFD-*1# was used in the following study (Supplementary Fig. 1). siRNA sequences were shown in Supplementary Table 1. Lipofectamine RNAiMAX transfection reagent (Invitrogen, Carlsbad, CA, USA) and Opti-MEN (Invitrogen, Carlsbad, CA, USA) were used to transfect siRNAs according to the manufacturer’s instructions.

### CRISPR/Cas9-mediated genome editing

CRISPR/Cas9 system was used to knock out MTHFD2 expression in A549 cells. Three different sgRNA against human *MTHFD2* were synthesized by Sangon Biotech (Shanghai, China) and listed in Supplementary Table 2. PolyJet (SignaGen Laboratories, Frederick, MD, USA) was used as the transfection reagent. Firstly, 293 T cells were seeded at a high density of 1.5*10^^5^ cells/well 24 h before transfection in six-well plates. The cells were co-transfected with a recombinant lentiviral vector LentiCRISPRv2 containing with packaging plasmids pVSVG and pMD2G. 48 h later, the supernatant of 293 T cells containing virus was collected and transfected into A549 cells. After the following 24 h, puromycin (400 ng/mL) (Solarbio, Shanghai, China) was continuously added to the media for another 5 days for selection of virus-transduced cells. Cells were sorted and transferred to ninety-six well plates at the density of one single cell per well. The successful MTHFD2-KO cell clone was tested by western blot analysis (Supplementary Fig. 2).

### miRNA mimics synthesis and transfection

miRNA mimics were synthesized by GenePharma (Shanghai, China) as follows: *hsa-miR-99a-3p* mimics: 5'-CAAGCUCGCUUCUAUGGGUCUG-3' and 5'-GACCCAUAGAAGCGAGCUUGUU-3'. Lipofectamine 2000 and DMEM were used to transfect miRNA mimics according to the manufacturer’s procedures.

### Cell proliferation assay

MTT assay was performed to explore cell proliferative abilities. Briefly, cells were incubated with 0.5 mg/mL 3-(4,5-dimethylthiazol-2-yl)-2,5-diphenyltetrazolium bromide (MTT) for 2 h at 37 °C (Solarbio, Shanghai, China). After incubation, cell supernatant was removed and the purple formazan crystals were dissolved by DMSO. The absorbance was measured at 570 nm using a Multiskan GO Microplate Reader (Thermo Fisher Scientific, Walthan, UA, USA).

### Flow Cytometry analysis

Cell apoptosis was detected by flow cytometry using a PI/Annexin V kit (BD Biosciences, Franklin Lakes, NJ, USA). In brief, cells were collected and resuspended in 1 × Binding Buffer followed by incubation with FITC-labeled Annexin V for 15 min in dark. PI was added to each sample 5 min before detection. Flow cytometry analysis was performed on BD FACS Canto II machine with FACS Diva software.

### JC-1 staining assay

JC-1 (Beyotime, Shanghai, China) immunofluorescence staining was used to assess the mitochondrial membrane potential (Δ*ψ*m), which reflected the early stage of apoptosis. The shift from aggregates (red) to monomers (green) fluorescence intensity is an indicative of depolarization of Δ*ψ*m. In brief, cells were exposed to JC-1 working solution for 30 min at 37 °C. Images were captured and analyzed using a fluorescence microscope (Zeiss, Oberkochen, Germany).

### Wound healing assay

Wound healing assay was performed to detect cell migration. In brief, cells were seeded in 12-well plates to reach 100 % confluence. Wounds were made by scratching cell layer using a sterile 200 μL micropipette tip. Then cells were further cultured in medium containing 1% FBS for 24 h. The wound areas were immediately captured using a light microscope (Zeiss, Oberkochen, Germany) and quantified by ImageJ software (v1.8.0, National Institutes of Health, Germany).

### RNA extraction and real-time quantitative PCR

Total RNA was isolated by TRIzol reagent (Invitrogen, Carlsbad, CA, USA). mRNA and miRNA were reversed transcribed into cDNA using RT Master Mix reagent (Takara, Shiga, Japan) and miRcute Plus miRNA First-Strand cDNA Kit (TIANGEN BIOTECH, Shanghai, China), respectively. qPCR amplification was performed in ABI Stepone Plus machine (Thermo Fisher Scientific, Walthan, UA, USA) with SYBR Green™ Premix Ex Taq™ II (Takara, Shiga, Japan) or miRcute Plus miRNA qPCR Kit (TIANGEN BIOTECH, Shanghai, China). Primers (synthesized by Sangon Biotech) used in this study were listed in Supplementary Table 3. Each mRNA level was normalized to *β-actin* gene expression. *miR-99a-3p* level was normalized to *U6* gene expression. Relative mRNA expression was calculated by the 2^-ΔΔCt^ method.

### Immunoblot and antibodies

Cell homogenates and tissues were lysed in lysis buffer (25 mmol/L pH 7.6 Tris-HCl, 150 mmol/L NaCl, 1% sodium deoxycholate and 1% NP-40) with protease inhibitor (Roche, Basel, Switzerland). 10 μg of protein sample was run on SDS-PAGE and transferred to 0.22 μm PVDF membranes. After blocking with 5% non-fat milk powder for 1 h, the membranes were incubated with primary antibodies overnight at 4 °C. Next day, all membranes were incubated with HRP-conjugated secondary antibodies for 90 min. The protein expression levels were visualized on Bio-rad ChemiDoc XRS^+^ Imaging Systems (Bio-rad, Hercules, CA, USA) using an ECL detection kit (Merck Millipore, Massachusetts, USA). Images were quantified by ImageJ software (v1.8.0, National Institutes of Health, Germany). The primary antibodies Zeb1 (#70512), E-cadherin (#3195), Vimentin (#5741), Snail (#3879), Cyclin D1 (#2978) and α-tubulin (#3873) were purchased from Cell Signaling Technology (Beverly, MA, USA). MTHFD2 (sc-100750), MCM4 (sc-29317) and MCM7 (sc-9966) were obtained from Santa Cruz Biotechnology (Dallas, TX, USA). β-actin (E021020) was purchased from EarthOX (Millbrae, CA, USA). The secondary antibodies goat anti-rabbit or goat anti-mouse IgG-HRP was obtained from Jackson ImmunoResearch Laboratories (West Grove, PA, USA). The detailed information of the antibodies can be found in Supplementary Table 4, and the original blots are provided in Supplementary Data.

### Immunohistochemistry

Immunohistochemistry was conducted to detect the expression of MTHFD2 in human lung cancerous tissues and paracancerous tissues. Briefly, paraffin embedded tissues were cut into 5 µm slides. Tissues were incubated with anti-MTHFD2 antibody (sc-100750, Santa Cruz Biotechnology, Dallas, TX, USA) overnight at 4 °C. Next day, each sample was incubated with HRP-labeled second antibodies (Servicebio, Wuhan, China) for 1 h. The expression level of MTHFD2 was analyzed using a light microscope (Zeiss, Oberkochen, Germany). Slides staining were scored according to intensity under light microscopy: 0 = background staining intensity; 1 = weak staining intensity; 2 = moderate staining intensity; 3 = strong staining intensity.

### Dual luciferase report assay

The putative binding sites that *miR-99a-3p* interacted with MTHFD2 at sites 1708-1714 of the 3' UTR and MCM4 at sites 96-102 of the 3' UTR were identified by TargetScan (http://www.targetscan.org/). Dual luciferase reporter assay was used to confirm the interaction between binding sites. Briefly, Human embryonic kidney 293 T cells were seeded into 24-well plates until reached 60% confluence before transfection. Then cells were co-transfected with 100 ng GP-miRGLO-MTHFD2-WT (or MUT) or GP-miRGLO-MCM4-WT (or MUT) reporter plasmids (designed and constructed by GenePharma) and 50 nM *miR-99a-3p* mimics (or *NC* mimics) using Lipofectamine 2000 reagent. After transfection for 48 h, the firefly and Renilla luciferase activities were measured by Dual Luciferase Reporter Assay kit (Promega, Madison, WA, USA) using a Varioskan LUX Multimode Microplate Reader (Thermo Fisher Scientific, Walthan, UA, USA).

### Bioinformatics data extraction and analysis

The gene expression data (535 tumor cases and 59 normal tissues, data type: HTSeq-FPKM), miRNA isoform expression data (483 tumor cases and 45 normal tissues), and corresponding clinical information of lung adenocarcinoma were downloaded and collected from TCGA-GDC (https://portal.gdc.cancer.gov/). The different expression analysis was visualized by boxplots and normal lung samples were excluded to conduct survival analysis. Both differential expression analysis and survival analysis were conducted using R (v.4.1.2). The relationship between target genes and *MTHFD2* as well as the expression of *miR-99a-3p* and its target genes were analyzed with the Spearman’s correlation. The cutoff of *MTHFD2* and *miR-99a-3p* expression were generated by the median value. R packages including *survival, survminer, limma, beeswarm* were used in this study.

### Statistical analysis

All quantitative data were analyzed using GraphPad Prism 6.0 software (GraphPad Software Inc, San Diego, CA, USA). Statistical significance was evaluated by unpaired *t*-test for comparing between two groups or one-way ANOVA for multiple groups. All data were presented as mean ± SD. A value of *P* < 0.05 was considered as the criterion of significance.

## Results

### MTHFD2 is highly expressed in LUAD patients and predicts an unfavorable prognosis

**Fig. 1:**
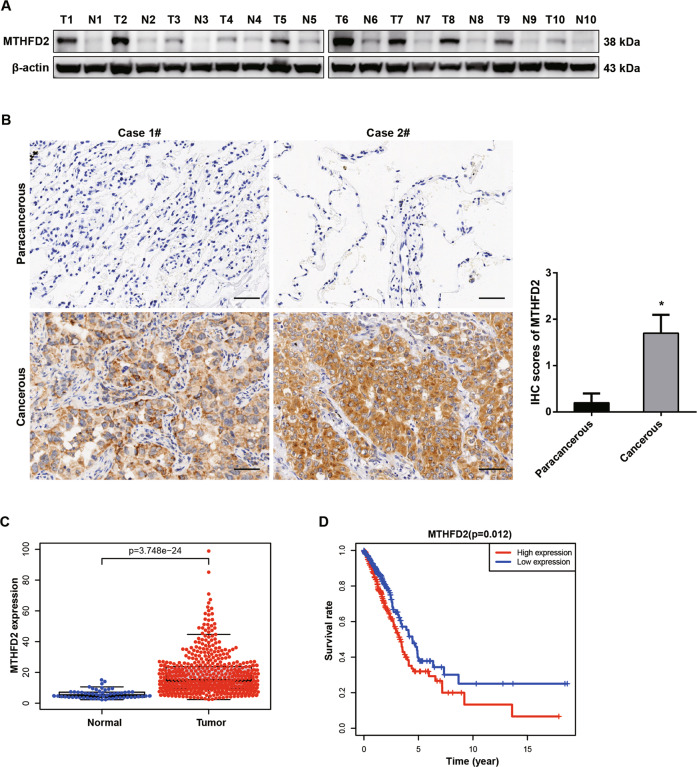
MTHFD2 is highly expressed in LUAD patients and predicts an unfavorable prognosis. **A** Western blot analysis of MTHFD2 protein expression in cancerous tissues (T) and matched paracancerous tissues (N) from LUAD patients (*n* = 10). **B** Representative IHC staining (left panel) and scores (right panel) of MTHFD2 expression in 10 paired cancerous and paracancerous tissues from LUAD patients. Scale bar, 50 μm. **C** Relative mRNA levels of MTHFD2 in tumor tissues (right panel, *n* = 535) and normal tissues (left panel, *n* = 59) based on the LUAD TCGA database. **D** Kaplan-Meier survival analysis based on different MTHFD2 gene expression levels in LUAD patients. *P < 0.05.

We first evaluated the expression of MTHFD2 protein in cancerous and matched paracancerous tissues in LUAD patients by immunoblot and immunohistochemistry assays. Total twenty pairs of tissues (ten for WB and ten for IHC, respectively) were used in this study. As shown in Fig. 1A, an increased MTHFD2 protein levels were obviously detected in LUAD cancerous tissues compared with that in matched paracancerous tissues. IHC analysis also confirmed that MTHFD2 protein expression was statistically higher in LUAD cancerous tissues than that in paracancerous tissues (Fig. 1B). Additionally, using bioinformatics data downloaded from TCGA databases, we investigated the MTHFD2 expression and prognosis in LUAD patients. Bioinformatics data suggested that the transcriptional level of *MTHFD2* was significantly increased in human LUAD tissues compared with normal lung tissues (Fig. 1C). The increased mRNA level of *MTHFD2* was correlated with an unfavorable prognosis (Fig. 1D). These results indicated that MTHFD2 was aberrantly upregulated in LUAD cancer and was closely correlated with a poor prognosis in LUAD patients.

### Knockdown of MTHFD2 inhibits cell proliferation, migration, and induces cell apoptosis in LUAD cell lines

**Fig. 2:**
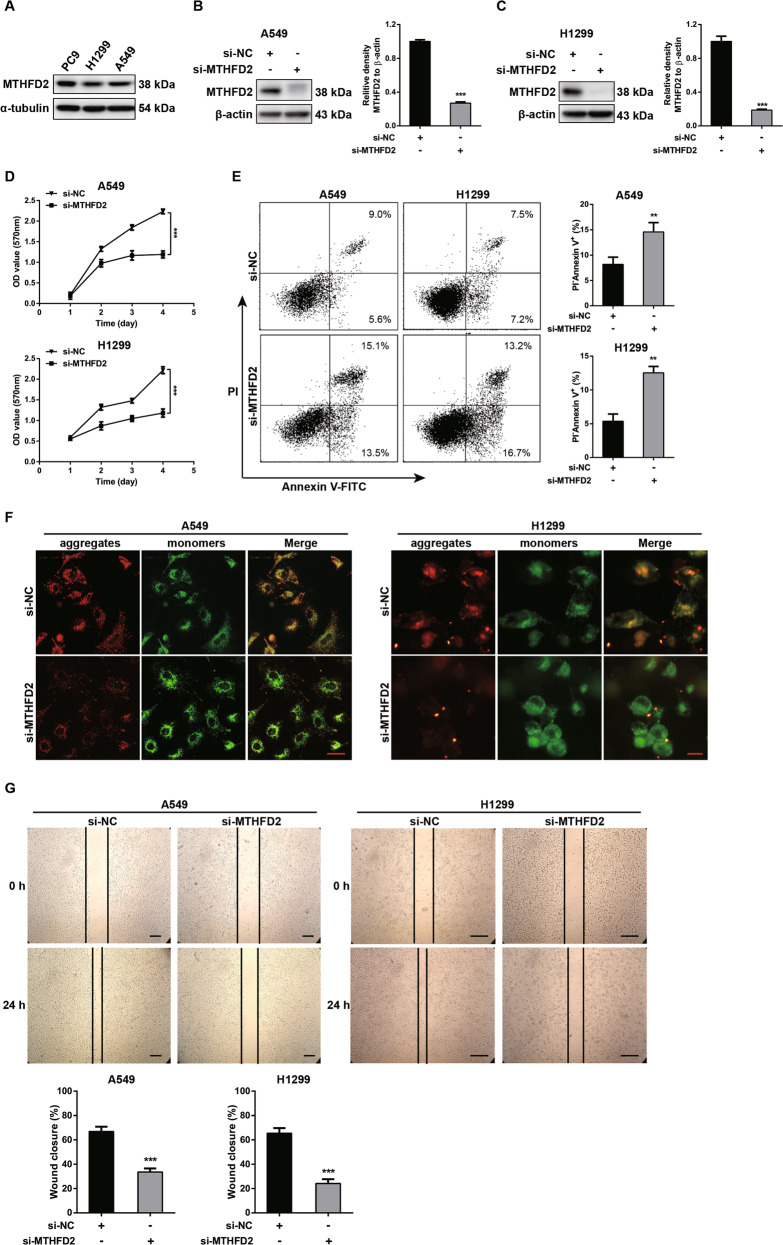
Knockdown of MTHFD2 inhibits cell proliferation, migration, and induces cell apoptosis in LUAD cell lines. **A** Western blot analysis of MTHFD2 expression in A549, H1299 and PC9 lung cancer cell lines. **B**, **C** Western blot analysis was conducted to confirm the knockdown efficiencies of MTHFD2 in A549 and H1299 cells. The quantification of MTHFD2 band density relative to β-actin was shown at the right histogram. **D** MTT assay examined the cell proliferative abilities at the indicated time point in MTHFD2-knockdown A549 (up panel) and H1299 (down panel) cells. **E** FACS analysis detected the cell apoptosis in MTHFD2-knockdown A549 (left panels) and H1299 cells (right panels). The percentages of cell apoptosis (PI^-^/Annexin V^+^) in A549 (up) and H1299 (down) cells were quantified at the right histograms. **F** JC-1 staining showed the mitochondrial membrane potential (Δ*ψ*m) of A549 (left) and H1299 (right) cells after MTHFD2 knockdown for 48 h. Scale bar, 50 μm. **G** Wound healing assay showed the migration abilities in MTHFD2-knockdown A549 (left) and H1299 (right) cells. Wound closures were quantified in the bottom. Scale bar, 200 μm. **P < 0.01, ***P < 0.001.

To clarify the role of MTHFD2 in lung cancer cells, we first explored the protein expression of MTHFD2 in several LUAD cell lines and detected evident MTHFD2 expression in A549, H1299 and PC9 cell lines (Fig. 2A). Then, using siRNAs, we transiently knocked down MTHFD2 in A549 and H1299 cell lines and measured cell proliferative abilities. Immunoblot results confirmed the knockdown efficiency of MTHFD2 in A549 and H1299 cell lines (Fig. 2B, C). Interestingly, MTT assay demonstrated that the proliferative activities of A549 and H1299 cells were significantly inhibited in si-MTHFD2 group compared with si-control group (Fig. 2D). Moreover, knockdown of MTHFD2 in A549 and H1299 cells induced an increased apoptosis compared with si-control group observed by flow cytometry analysis (Fig. 2E). To further confirm MTHFD2-knockdown induced cell earlier apoptosis, mitochondrial membrane potential (Δ*ψ*m) was evaluated by using fluorescent dye JC-1. Compared with si-control group, a reduced mitochondrial membrane potential was observed in si-MTHFD2 group (Fig. 2F). To investigate the effect of MTHFD2-knockdown on cell migration, wound healing assay was performed and data showed impaired cell migration abilities in si-MTHFD2 lung cell lines compared with controls (Fig. 2G). These results demonstrated that MTHFD2-knockdown inhibited the LUAD cell proliferation and migration, and induced the apoptosis both in A549 and H1299 cells.

### CRISPR/Cas9-mediated MTHFD2 deletion on A549 cell proliferation, apoptosis and migration

**Fig. 3:**
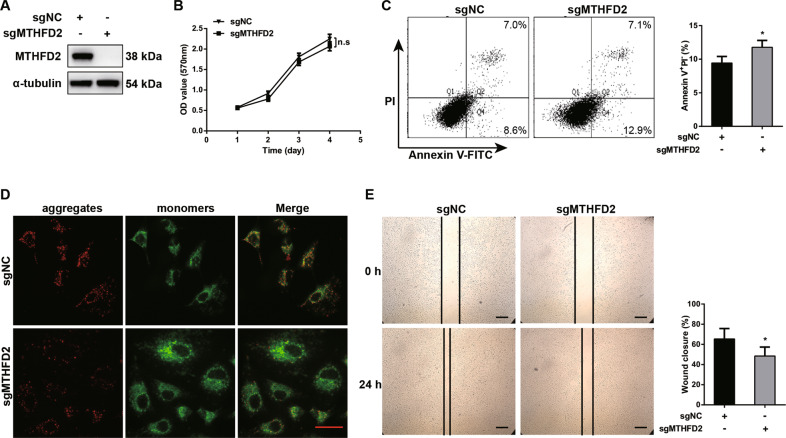
CRISPR/Cas9-mediated MTHFD2 deletion on A549 cell proliferation, apoptosis and migration. **A** Western blot confirmed the knockout efficiency of MTHFD2 in A549 cells transfected with the sgRNA and negative control. **B** MTT assay showed the cell proliferation in MTHFD2-knockout cells. **C** FACS analysis detected the cell apoptosis in MTHFD2-knockout A549 cells. The percentages of cell apoptosis (PI^-^/Annexin V^+^) were quantified at the right histogram. **D** JC-1 staining showed the mitochondrial membrane potential (Δ*ψ*m) in MTHFD2-knockout A549 cells. Scale bar, 50 μm. **E** Wound healing assay showed the migration in MTHFD2-knockout A549 cells. Wound closure was quantified at the right histogram. Scale bar, 200 μm. n.s: no significant difference, *P < 0.05.

In the following studies, we further generated a stable MTHFD2-knockout cell line using the CRISPR/Cas9 genome editing technique and examined the cell proliferation, apoptosis and migration. A clear lack of MTHFD2 expression is seen for single deleted A549 cells by western blot (Fig. 3A). However, no significant difference of cell proliferative activity was detected in MTHFD2-knockout A549 cells compared with controls (Fig. 3B), which was inconsistent with the previous result of transient knockdown of MTHFD2 in A549 cells (Fig. 2D). Nevertheless, Annexin V/PI analysis by Flow cytometry and mitochondrial membrane potential detection by JC-1 staining confirmed that increased apoptosis was observed in MTHFD2 deletion lung cancer cells (Fig. 3C, D). Additionally, wound healing assay showed that MTHFD2 deletion significantly decreased cell migration (Fig. 3E). These data confirmed that suppressed cell migration and increased cell apoptosis were seen in MTHFD2-knockout lung cancer cells.

### Downstream molecular targets in MTHFD2-knockdown LUAD cell lines

**Fig. 4:**
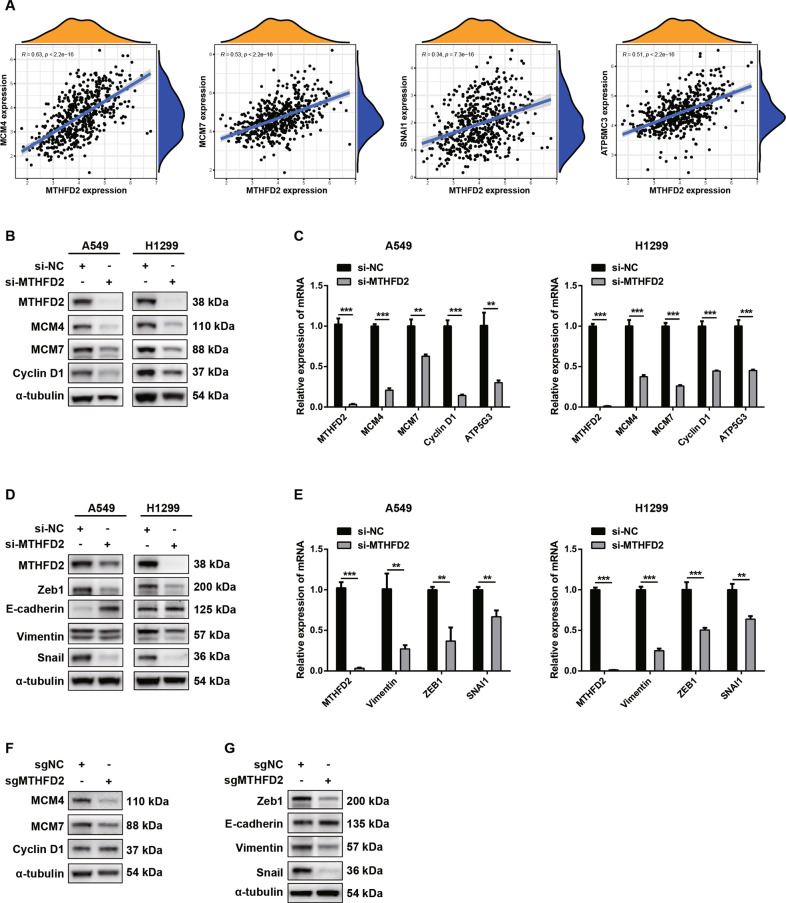
Downstream molecular targets in MTHFD2-knockdown LUAD cell lines. **A** TCGA database showed the positive correlation between *MTHFD2* and *MCM4*, *MCM7*, *SNAI1* and *ATP5MC3*. **B** The protein levels of MTHFD2, MCM4, MCM7, Cyclin D1 in MTHFD2-knockdown A549 (left panels) and H1299 (right panels) cells. α-tubulin was used as an internal control. **C** The mRNA levels of *MTHFD2*, *MCM4*, *MCM7*, *Cyclin D1* and *ATP5G3* normalized to *β-actin* in MTHFD2-knockdown A549 (left histogram) and H1299 (right histogram) cells. **D** The protein levels of MTHFD2, Zeb1, E-cadherin, Vimentin and Snail in MTHFD2-knockdown A549 (left panels) and H1299 (right panels) cells. α-tubulin was used as an internal control. **E** The mRNA levels of *MTHFD2*, *Vimentin, ZEB1 and SNAI1* normalized to *β-actin* in MTHFD2-knockdown A549 (left histogram) and H1299 (right histogram) cells. **F**, **G** The protein levels of cell growth regulators (MCM4, MCM7, Cyclin D1) and EMT markers (Zeb1, E-cadherin, Vimentin, Snail) in MTHFD2-knockout A549 cells. α-tubulin was used as an internal control. **P < 0.01, ***P < 0.001.

To further explore the fundamental roles of MTHFD2 during LUAD progression, we conducted series of studies to identify the downstream targets of MTHFD2 correlating cell proliferation, apoptosis and migration. Firstly, we performed bioinformatic studies to investigate the correlations of core gene expressions involving cell growth and EMT with MTHFD2 using LUAD samples from TCGA database. Interestingly, the expression of *MTHFD2* was positively correlated with the expressions of *MCM4*, *MCM7*, *ATP5MC3*, and *SNAI1* (Fig. 4A).

Then, we further clarified the gene and protein expressions of these MTHFD2-related molecules in MTHFD2-knockdown A549 and H1299 cells. As shown in Fig. 4B, the protein levels of MCM4, MCM7, and Cyclin D1 were significantly reduced in MTHFD2-knockdown A549 and H1299 cells. Correspondingly, the significant decreased transcriptional levels of *MCM4*, *MCM7*, *Cyclin D1*, and *ATP5G3* were also observed in MTHFD2-knockdown A549 and H1299 cells (Fig. 4C). Additionally, the protein and mRNA levels of EMT correlated markers including Zeb1, Vimentin and Snail were down-regulated, while the protein level of E-cadherin was upregulated in MTHFD2-knockdown A549 and H1299 cells (Fig. 4D, E). Moreover, the expressions of cell growth regulators MCM4 and MCM7 were decreased as well as EMT correlated markers Zeb1, Vimentin and Snail were reduced in MTHFD2-knockout A549 cells (Fig. 4F, G). Therefore, we deduced that MTHFD2 promoted LUAD cells proliferation and migration through regulating cell growth and EMT process.

### *miR-99a-3p* serves as a direct upstream regulator of MTHFD2 in LUAD cells

**Fig. 5:**
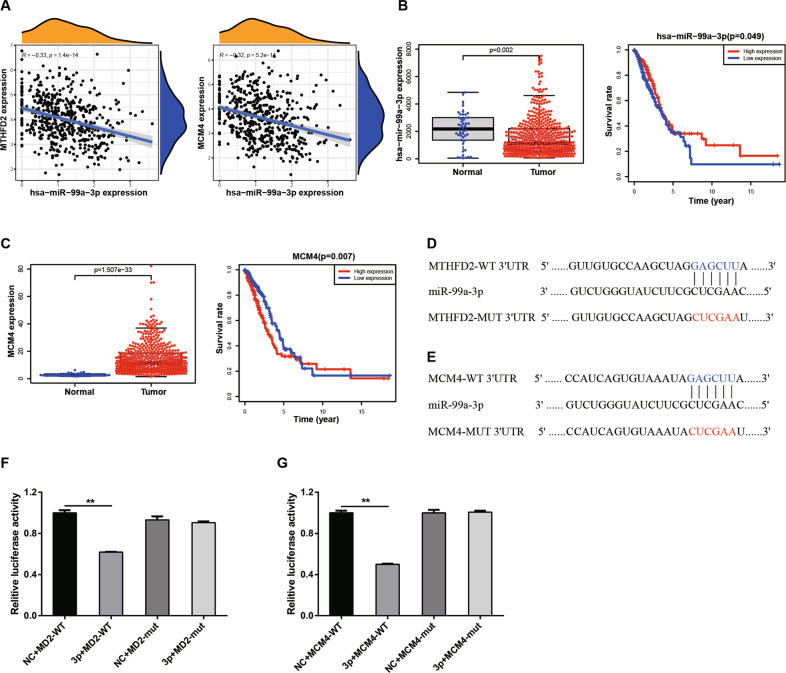
*miR-99a-3p* serves as a direct upstream regulator of MTHFD2 in LUAD cells. **A** TCGA database showed the negative correlation between *miR-99a-3p* and *MTHFD2* and *MCM4*. **B** The relative mRNA levels of *miR-99a-3p* in LUAD tissues (*n* = 483) and normal tissues (*n* = 45) based on the TCGA database. Kaplan–Meier survival analysis based on different *miR-99a-3p* expression levels in LUAD patients was shown at the right panel. **C** The relative mRNA levels of *MCM4* in LUAD tissues (*n* = 535) and normal tissues (*n* = 59) based on the TCGA database. Kaplan–Meier survival analysis based on different MCM4 expression levels in LUAD patients was shown at the right panel. **D**, **E** The putative binding sites for *miR-99a-3p* in 3' UTR of MTHFD2 and MCM4. **F**, **G** Dual luciferase reporter assay indicated the luciferase activities of MTHFD2-WT/MUT and MCM4-WT/MUT using *NC* mimics or *miR-99a-3p* mimics overexpressed A549 and H1299 cells. **P < 0.01.

Since dysregulation of miRNAs plays a crucial role in lung tumorigenesis, we next explored the potential upstream regulator mediating MTHFD2 expression and function in LUAD. Firstly, we analyzed the TCGA LUAD data again and found that *miR-99a-3p* was conversely correlated with *MTHFD2* and *MCM4* expressions (Fig. 5A). In addition, the expression of *miR-99a-3p* was significantly down-regulated in human LUAD tumor tissues compared with normal controls, and moreover, low expression of *miR-99a-3p* was associated with poor prognosis in LUAD patients using TCGA database (Fig. 5B). Furthermore, the expression of *MCM4* was significantly upregulated in human LUAD tumor tissues compared with normal controls, and higher expression of *MCM4* predicted poor prognosis in LUAD patients (Fig. 5C). Thus, we hypothesized that *miR-99a-3p* may be associated with regulating MTHFD2 and MCM4 in LUAD.

To understand the potential mechanisms by which *miR-99a-3p* regulated MTHFD2 and MCM4 expressions, we used TargetScan (http://www.targetscan.org/) and identified putative binding sites for *miR-99a-3p* in 3' UTR of MTHFD2 and MCM4 (Fig. 5D, E). To further confirm the direct interaction between *miR-99a-3p* and MTHFD2/MCM4, we constructed WT and mutant 3' UTR of MTHFD2/MCM4 in a commercially available plasmid. Dual luciferase report assays showed that transfection of *miR-99a-3p* mimics significantly reduced the luciferase activities in MTHFD2-WT and MCM4-WT-transfected 293 T cells but did not affect that in MTHFD2-MUT and MCM4-MUT-transfected cells (Fig. 5F, G).

**Fig. 6:**
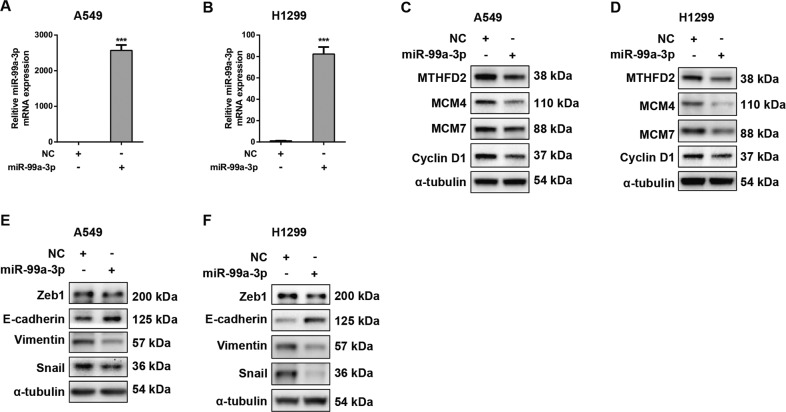
Overexpression of *miR-99a-3p* regulates cell growth and EMT associated proteins in LUAD cell lines. **A**, **B** qRT-PCR was conducted to confirm the overexpression efficiencies of *miR-99a-3p* in A549 and H1299 cells. **C**, **D** The protein levels of MTHFD2, MCM4, MCM7 and Cyclin D1 in *miR-99a-3p* overexpressed A549 and H1299 cells. α-tubulin was used as an internal control. **E**, **F** The protein levels of Zeb1, E-cadherin, Vimentin and Snail in *miR-99a-3p* overexpressed A549 and H1299 cells. α-tubulin was used as an internal control. ***P < 0.001.

Since *miR-99a-3p* directly regulated MTHFD2 and MCM4 expression, we further explored the role of *miR-99a-3p* in cell growth and EMT process in A549 and H1299 cells. The mRNA level of *miR-99a-3p* was dramatically upregulated after transfection of *miR-99a-3p* into in A549 and H1299 cells (*P* < 0.05) (Fig. 6A, B). Notably, *miR-99a-3p* overexpression decreased the protein levels of MTHFD2 and its downstream targets including MCM4, MCM7 and Cyclin D1 in A549 and H1299 cells (Fig. 6C, D). Moreover, Zeb1, Vimentin, and Snail expression levels were prominently down-regulated while E-cadherin expression was upregulated in *miR-99a-3p*-overexpressed A549 and H1299 LUAD cells (Fig. 6E, F). Together, the results suggested that *miR-99a-3p* targeted 3' UTR regions of MTHFD2 and MCM4, and therefore mediated their functions directly in LUAD cells.

### Specific inhibition of MTHFD2 function improves anti-tumor sensitivities induced by pemetrexed in LUAD

**Fig. 7:**
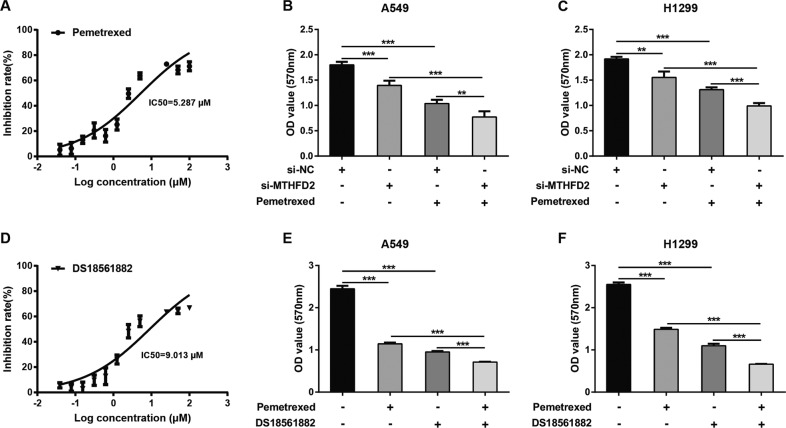
Specific inhibition of MTHFD2 function improves anti-tumor sensitivities induced by pemetrexed in LUAD. **A** The IC50 value of pemetrexed. **B**, **C** A549 and H1299 cells transfected with si-NC or si-MTHFD2 were co-cultured with or without pemetrexed (5 μM) for 72 h. Cell proliferative abilities were detected by MTT assay. **D** The IC50 value of MTHFD2 inhibitor DS18561882. **E**, **F** A549 and H1299 cells were treated with pemetrexed (5 μM), DS18561882 (10 μM), or combination for 5 days. Cell proliferative abilities were detected by MTT assay. **P < 0.01, ***P < 0.001.

Pemetrexed is the first-line chemotherapeutic drugs for LUAD patients. To explore whether targeting MTHFD2 together with pemetrexed can improve therapeutic advantages in LUAD, a specific MTHD2 inhibitor, DS18561882, was used in the following studies. At first, the concentrations of pemetrexed were selected ranging from 0.039 to 50 μM, and the IC50 value was 5.287 μM (Fig. 7A). MTT assays were conducted to compare cell viability after incubation of pemetrexed (5 μM) between MTHFD2-knockdown A549 cells and control cells. The repeated experiments also conducted in H1299 and control cells. The decreased cell viabilities induced by pemetrexed were detected both in MTHFD2-knockdown A549 and H1299 cells (Fig. 7B, C). Subsequently, we used MTHFD2 inhibitor DS18561882 to treat LUAD cells together with pemetrexed (5 μM). The concentrations of DS18561882 were ranging from 0.039 to 50 μM, and the IC50 value was 9.013 μM (Fig. 7D). Our result showed either DS18561882 or pemetrexed treatment led to a reduced cell viability compared with control groups both in A549 and H1299 cells (Fig. 7E, F). Interestingly, a combination of DS18561882 and pemetrexed exhibited synergistically antitumor activities compared to use a single agent, either DS18561882 or pemetrexed alone, both in A549 and H1299 cells (Fig. 7E, F). These data indicated that targeting MTHFD2 together with pemetrexed increased the antitumor sensibilities and provided therapeutic advantages in the treatment of LUAD cells.

## Discussion

Metabolic remodeling is the fundamental molecular feature of malignant tumors. Cancer cells require mitochondrial one-carbon units supporting their high proliferative rate, and therefore one-carbon pathway is centered in the metabolic abnormalities in many cancers [17, 21]. MTHFD2, an essential enzyme in this one carbon metabolic pathway which is upregulated in various cancer cells including LUAD, plays important roles in cancer cells growth and is responsible for poor prognosis of many cancer types [22].

In our studies, we showed that MTHFD2 was also highly expressed in cancerous tissues compared with paracancerous tissues in LUAD. Moreover, bioinformatics studies showed higher transcriptional levels of MTHFD2 were present in tumor tissues and correlated with poor survival rate in patients with LUAD. These findings are in agreement with previous reports that MTHFD2 acts as an oncogene to promote the development in lung cancer [23, 24]. Mechanism studies indicate that MTHFD2 serves as a rate limiting enzyme providing sufficient purine nucleotides which are DNA precursors required by the rapid growth of cancer cells [25]. Thus, we explored several molecules involving in DNA replication as the promising downstream targets of MTHFD2. Indeed, DNA helicases such as minichromosome maintenance protein family members, MCM4 and MCM7, which function as essential for eukaryotic chromosome replication, are positively correlated with the expression of MTHFD2 using by analyzing TCGA LUAD database. Transient silencing of MTHFD2 influences primary cellular events such as proliferation, apoptosis and migration by inhibiting several downstream molecules, including MCM4, MCM7, Cyclin D1, and ATP5G3, as well as Zeb1, Vimentin and Snail, which contribute the cancer cell growth and epithelial-to-mesenchymal transition (EMT).

The highly conserved MCM 2–7 heterohexameric complex is the core components of the replicative helicase and plays central roles in initiation and elongation of DNA replication [26]. MCM4 binds and unwinds various fork and extension structures carrying a single-stranded 3'-tail DNA [27]. MCM4 has been pointed out to be a gene related to poor prognosis of LUAD via bioinformatical analysis [28]. MCM4 is highly expressed in LUAD and knockdown MCM4 leads to suppressed growth in the lung cancer cells [29]. Our bioinformatics studies were also confirmed these findings. In addition to DNA helicases, we also observed decreased Cyclin D1 protein, a cell cycle regulator, in MTHFD2 deficient lung cancer cells. High expression of Cyclin D1 accelerates cell cycle in a variety of malignancies and contributes tumorigenesis [30, 31]. We also found that knockdown of MTHFD2 decreased ATP5G3 expression, which could affect the synthesis of ATP and ATP-dependent processes. Therefore, our data suggested that the over-activating one-carbon metabolic machinery may contribute the high proliferative event in cancer cells. Interestingly, in our stable MTHFD2 gene knockout lung cancer cell line using a CRISPR/Cas9 gene-editing technology, we did not observe proliferative difference between MTHFD2 deficient cells compared with wide type cells, but reduced gene expression was similarly as transient MTHFD2 knockdown, suggesting that cells may adapt to the low expression levels of MTHFD2 via using other alternate pathways rather than the mitochondrial pathway to compensate the growth and survive of cancer cells.

Additionally, a previous study suggested that loss of MTHDF2 is associated with EMT via regulating Wnt/β-catenin signaling and driving tumor growth and metastasis in LUAD [32]. EMT is mainly mediated by EMT-activating transcription factors including SNAIL (also SNAI1) and SLUG (also SNAI2), the basic helix–loop–helix factors TWIST (TWIST) and the zinc finger E-Box-binding homeobox factors (ZEB1 and ZEB2) [33]. All these transcription factors share the ability to repress the expression of epithelial genes and are likely to maintain stemness properties, linking them to the concept of cancer stem cells (CSCs). Overexpression of MTHFD2 was indicated in gefitinib resistant LUAD and associated with cancer stem-like properties [23]. In our study, we also observed the migration activity was impaired in MTHFD2-deficient lung cancer cells by affecting gene and protein expression of Zeb1, Vimentin and Snail.

We further conducted bioinformatics analysis to decipher promising upstream post-transcriptional regulators of MTHFD2. We revealed a negative correlation between *miR-99a-3p* and MTHFD2 at transcriptional levels in LUAD using the TCGA database. Overexpression of *miR-99a-3p* reduced the gene and protein expression of MTHFD2 as well as its downstream targets including MCM4, MCM7, Cyclin D1, Zeb1 and Snail. Luciferase reporter assay demonstrated that *miR-99a-3p* directly targeted 3' UTR regions of MTHFD2 and MCM4. Thus *miR-99a-3p*, MTHFD2 and MCM4 formed a network leading to regulate folic acid metabolism in LUAD. The activation of *miR-99a-3p* signaling cascades, at least partially, inhibited MTHFD2 expression in the one-carbon metabolic pathway in which may further interrupt DNA replication. *miR-99a-3p* is the passenger strand pre-*miR-99a*. In general theory, the passenger strand of a miRNA derived from a pre-miRNA has no biological function, however, accumulating evidence suggested that *miR-99a-3p* expression was significantly reduced in LUAD, head and neck squamous cell carcinoma (HNSCC) and prostate cancer, and may act as a tumor suppressor [34, 35].

Pemetrexed, a folate antagonist, is the first-line chemotherapeutic drugs, frequently used in the treatment of LUAD [36]. Pemetrexed suppresses core enzymes required for synthesizing DNA precursors and plays anti-tumor activates in patients with LUAD [37]. However, decreased response to pemetrexed happened after a period of drug administration due to drug resistance. Therefore, we tried to answer an important question that whether specific inhibition of MTHFD2 function by siRNA or a chemical compound can improve anti-tumor sensitivities induced by pemetrexed in LUAD. Indeed, increased antitumor efficacies induced by pemetrexed were observed in a MTHFD2 deficient cell line, indicating that targeting MTHFD2 together with pemetrexed provides therapeutic advantages in the treatment of LUAD. Therefore, we further explored the anti-tumor effects of a MTHFD2 inhibitor, DS18561882 at 10 µM in lung cancer cells. We showed that combined with DS18561882 and pemetrexed exhibited synergistically anti-tumor activities in lung cancer cells. These results provide extra evidence that targeting mitochondrial DNA synthesis pathway is a promising strategy in the treatment of LUAD.

Taken together, our results demonstrate the underlying molecular mechanisms of MTHFD2 in regulating proliferation and identify a novel therapeutic strategy improving the treatment efficacies in LUAD.

### Reporting summary

Further information on research design is available in the Nature Research Reporting Summary linked to this article.

## Supplementary information

Supplemental Figure 1

Supplemental Figure 2

Supplemental Tables

Supplemental Figure and Table legends

Original western blots

Reporting Summary

## Data Availability

The data in this study are available from the corresponding author upon reasonable request.
