# Supplementary figures and images for: Targeting mitochondrial one-carbon enzyme MTHFD2 together with pemetrexed confers therapeutic advantages in lung adenocarcinoma
Source: Cell Death Discov. 2022 Jul 5;8:307. doi: 10.1038/s41420-022-01098-y (PMC9256677; doi:10.1038/s41420-022-01098-y)

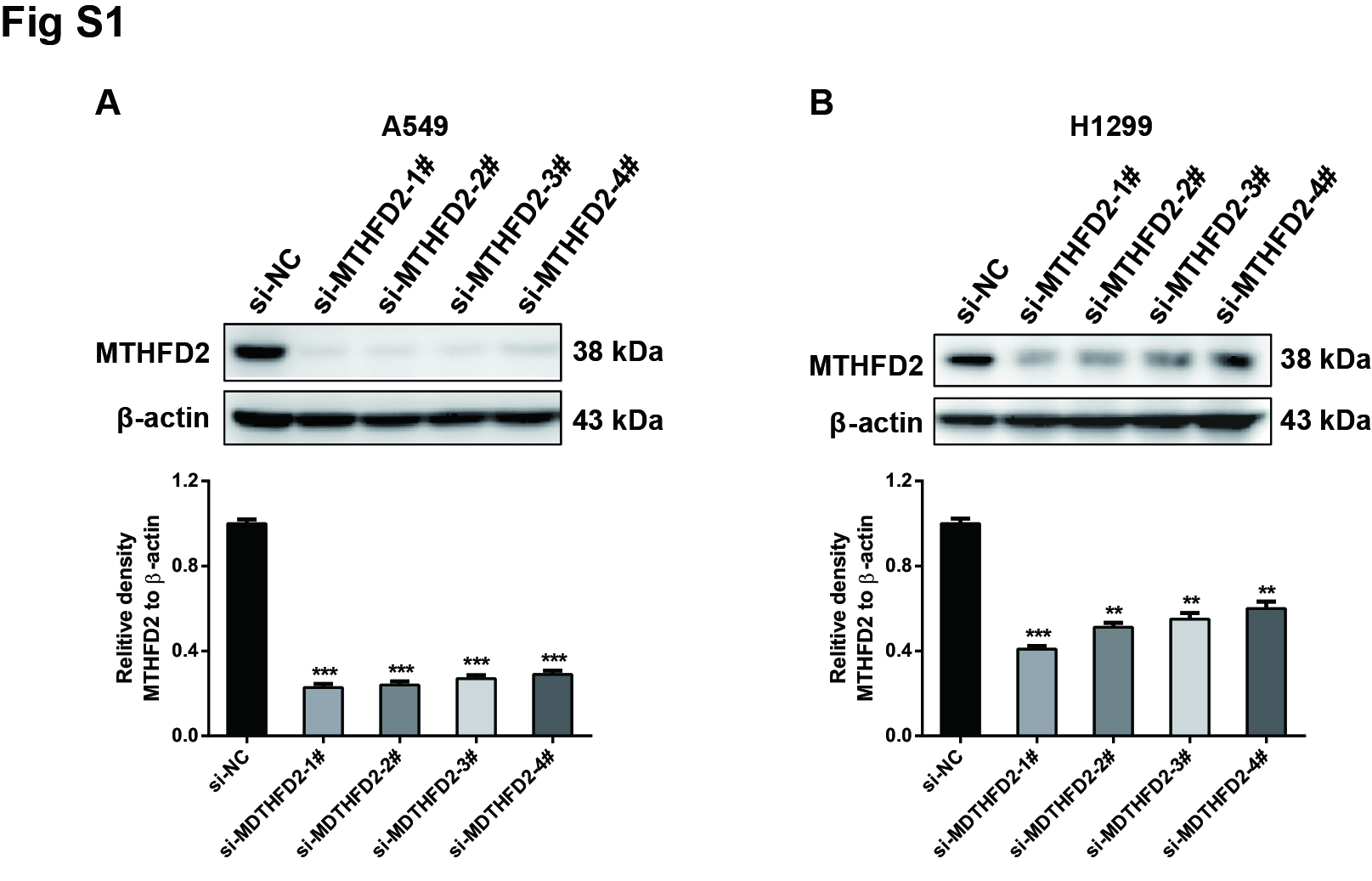

Supplement: Supplementary file 1 — Supplemental Figure 1 [file 41420_2022_1098_MOESM1_ESM.tif]

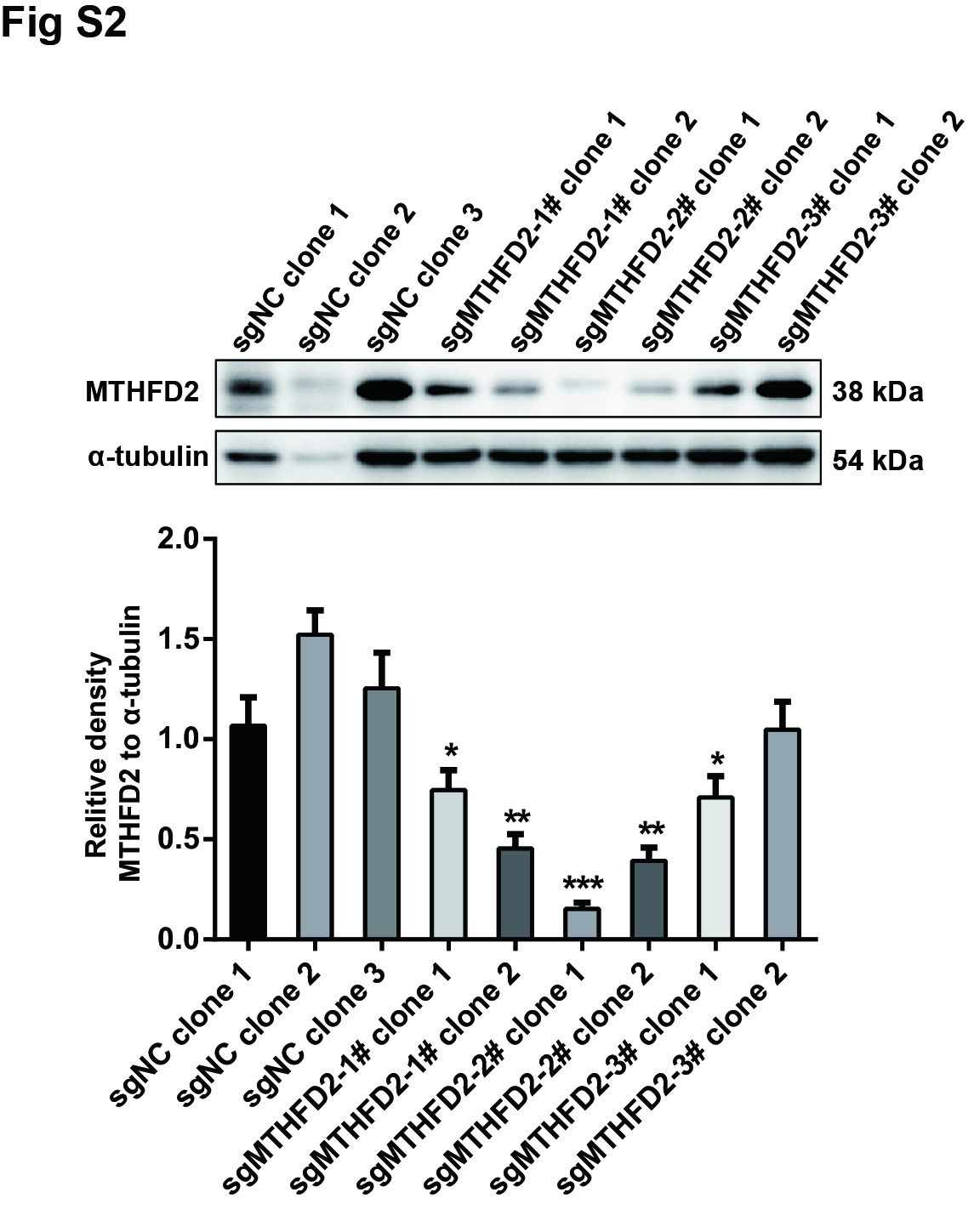

Supplement: Supplementary file 2 — Supplemental Figure 2 [file 41420_2022_1098_MOESM2_ESM.tif]

**Fig 1A**

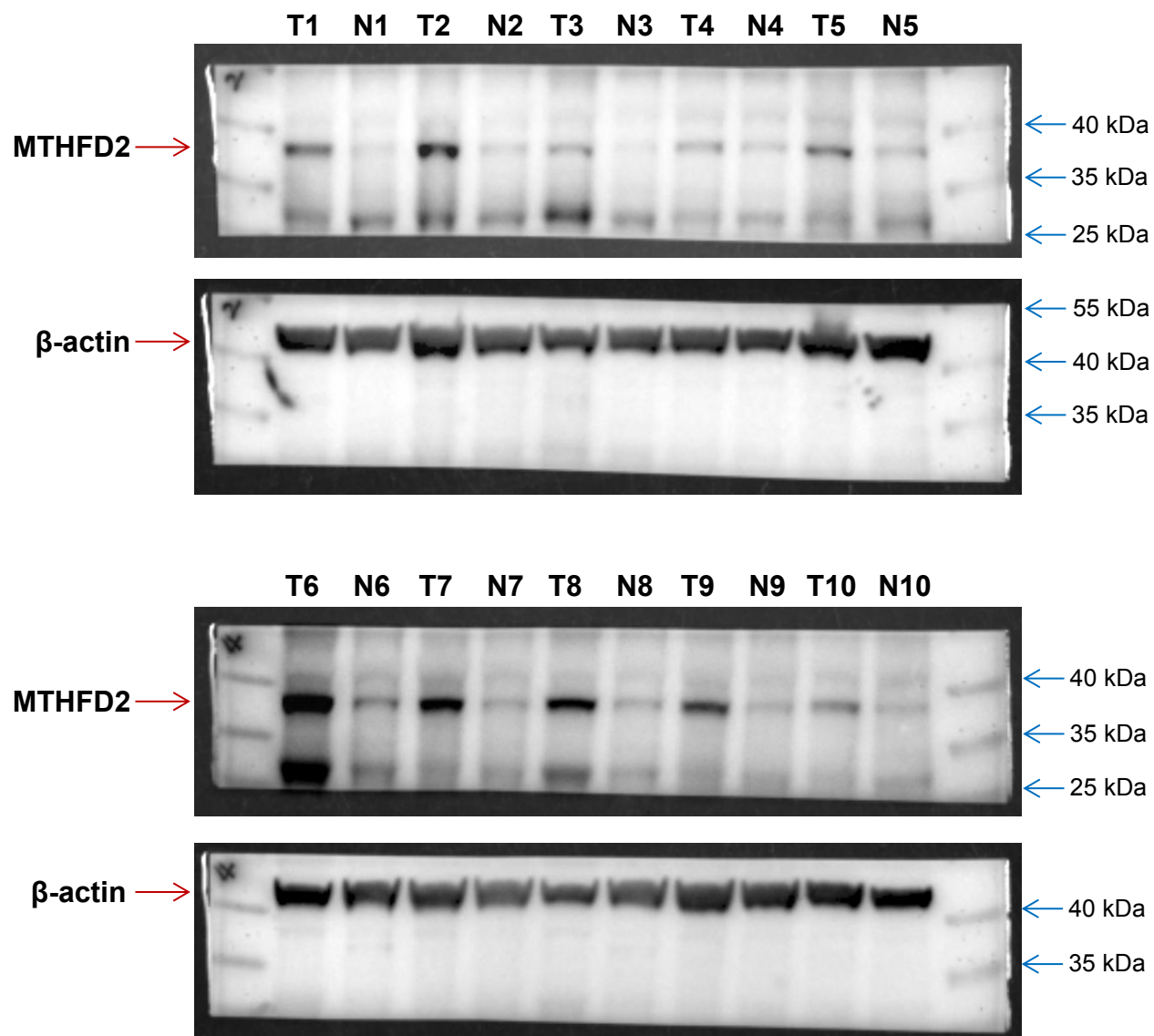

**Fig 2A**

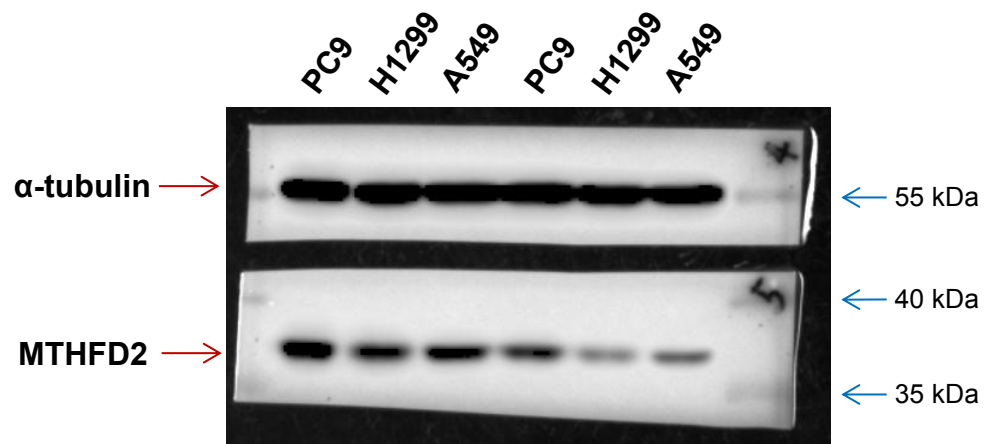

**Fig 2B**

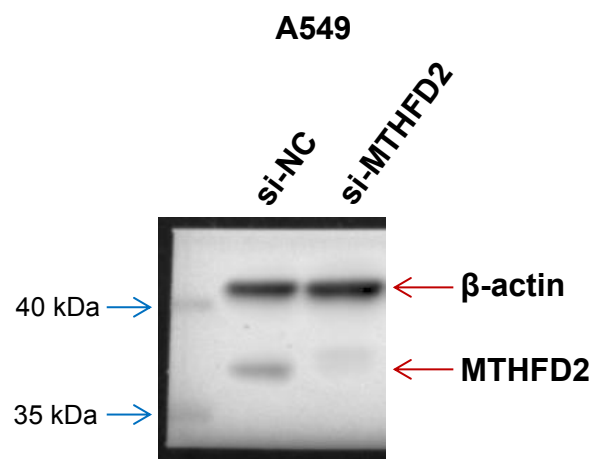

**Fig 2C**

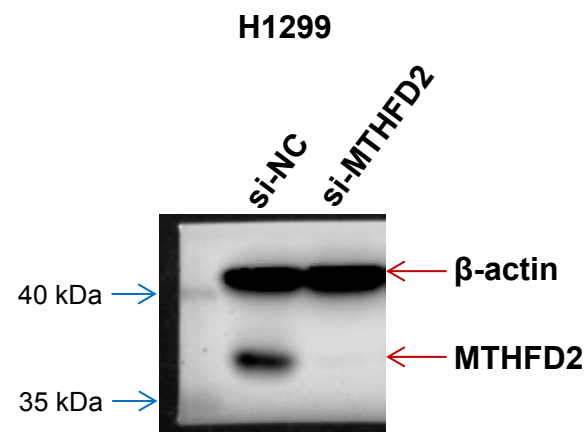

**Fig 3A**

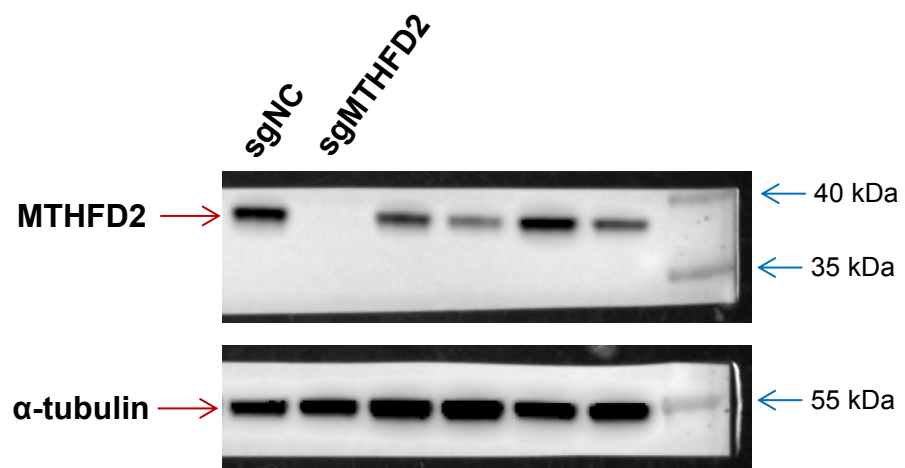

**Fig 4B**

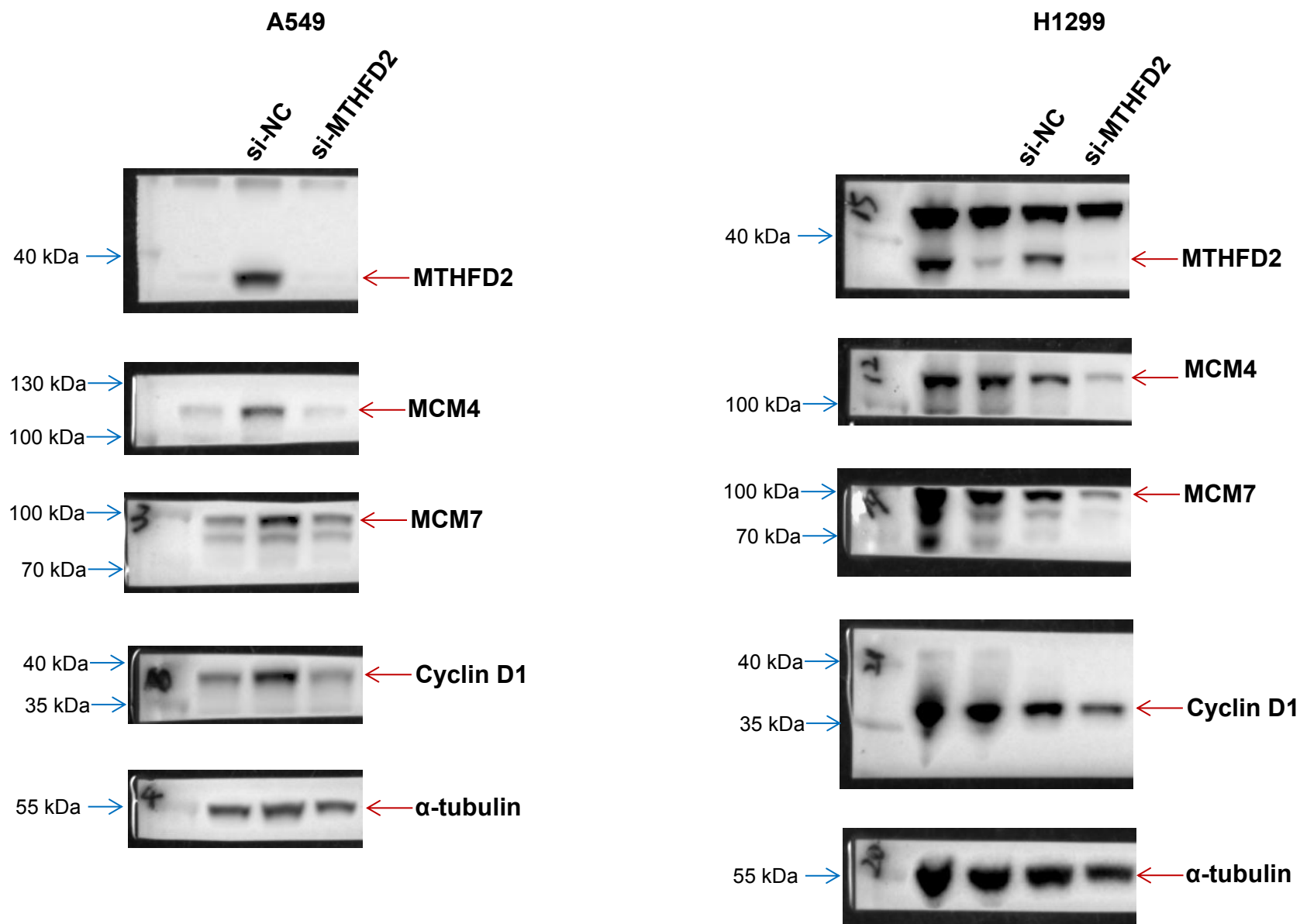

**Fig 4D**

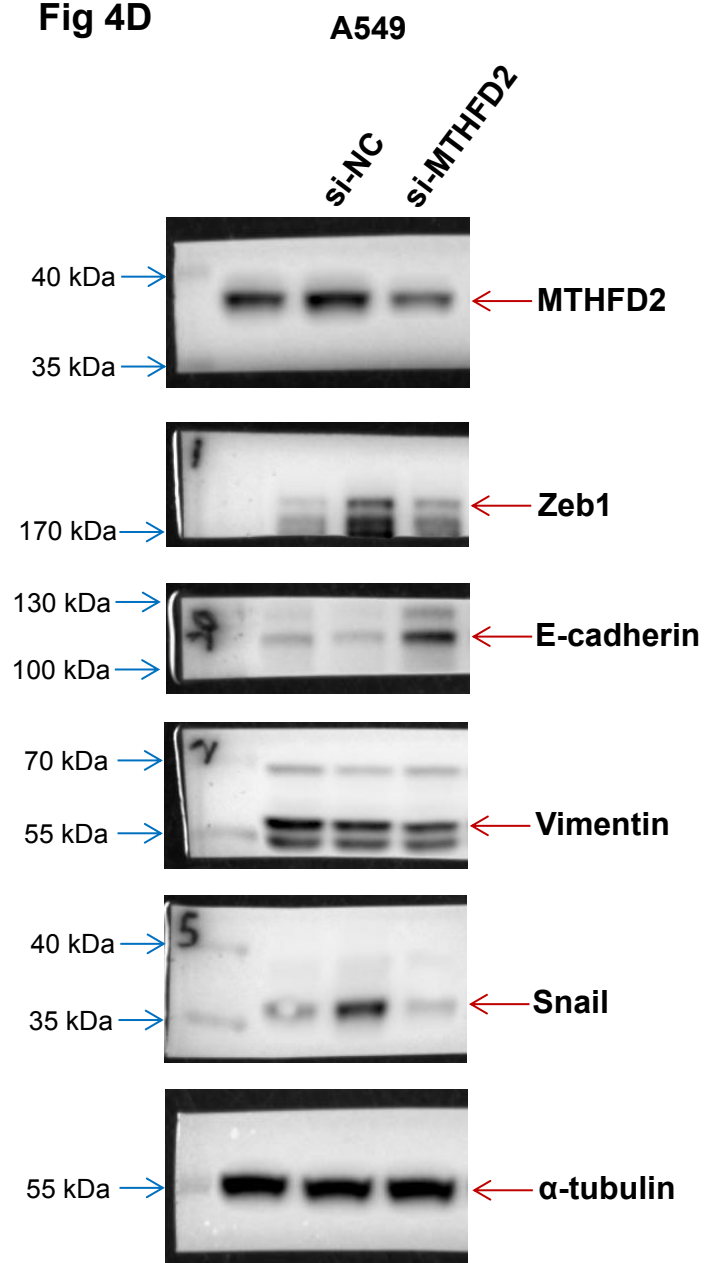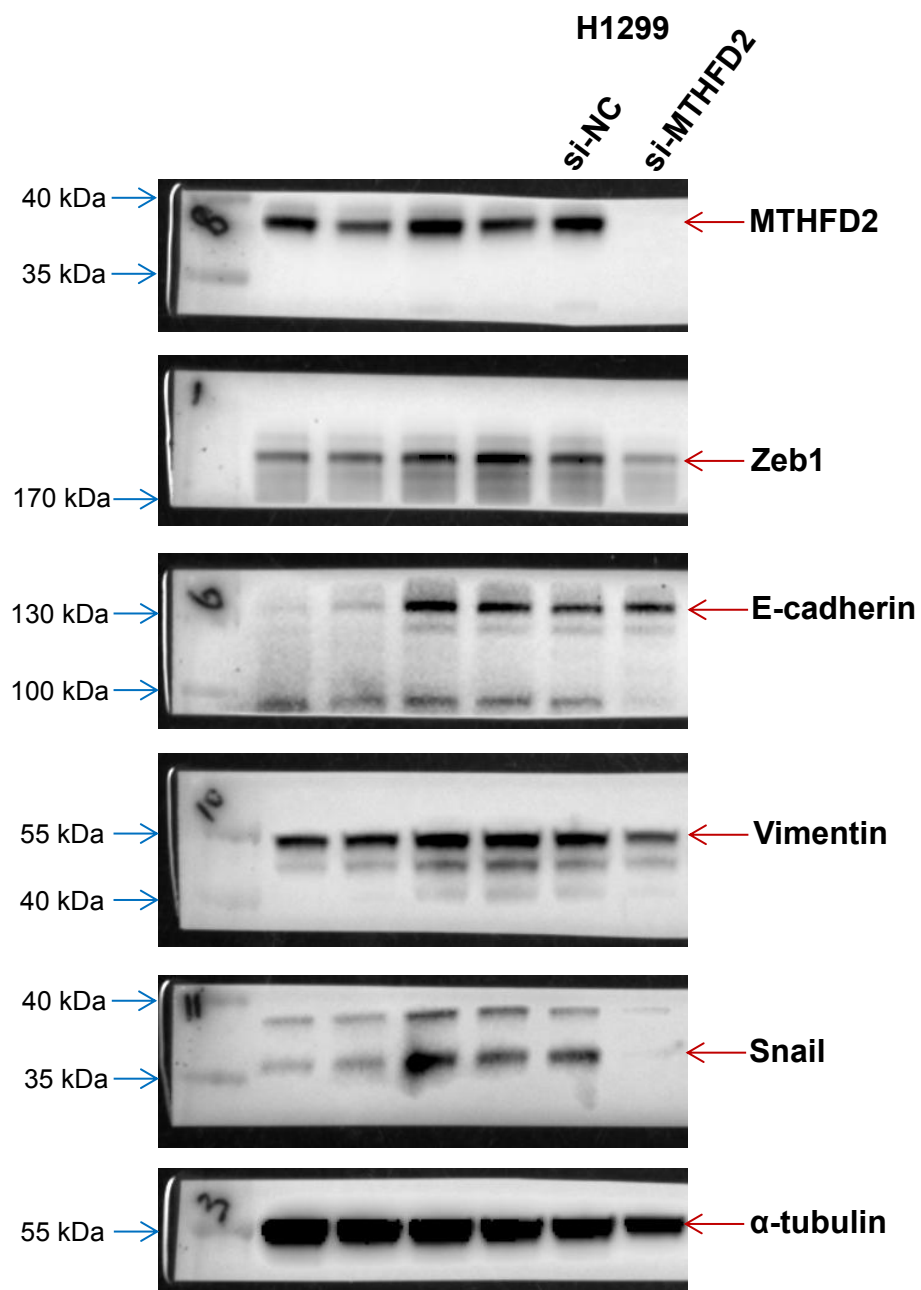

**Fig 4F**

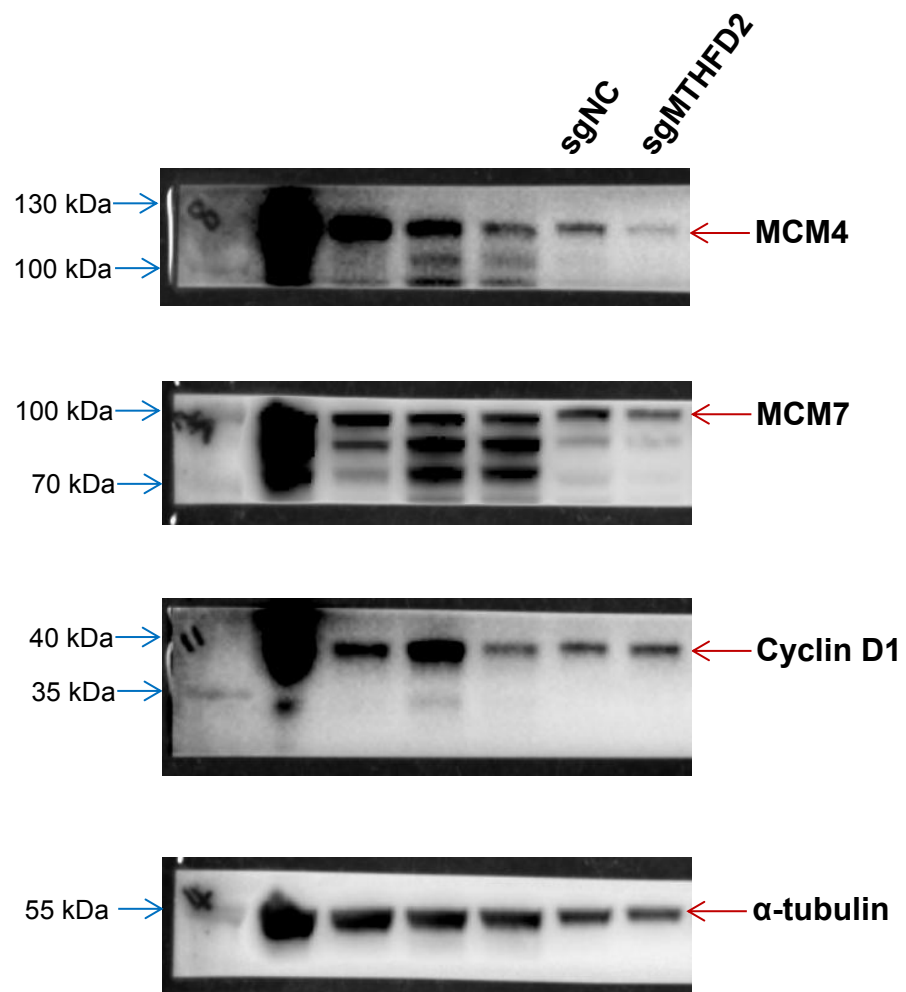

**Fig 4G**

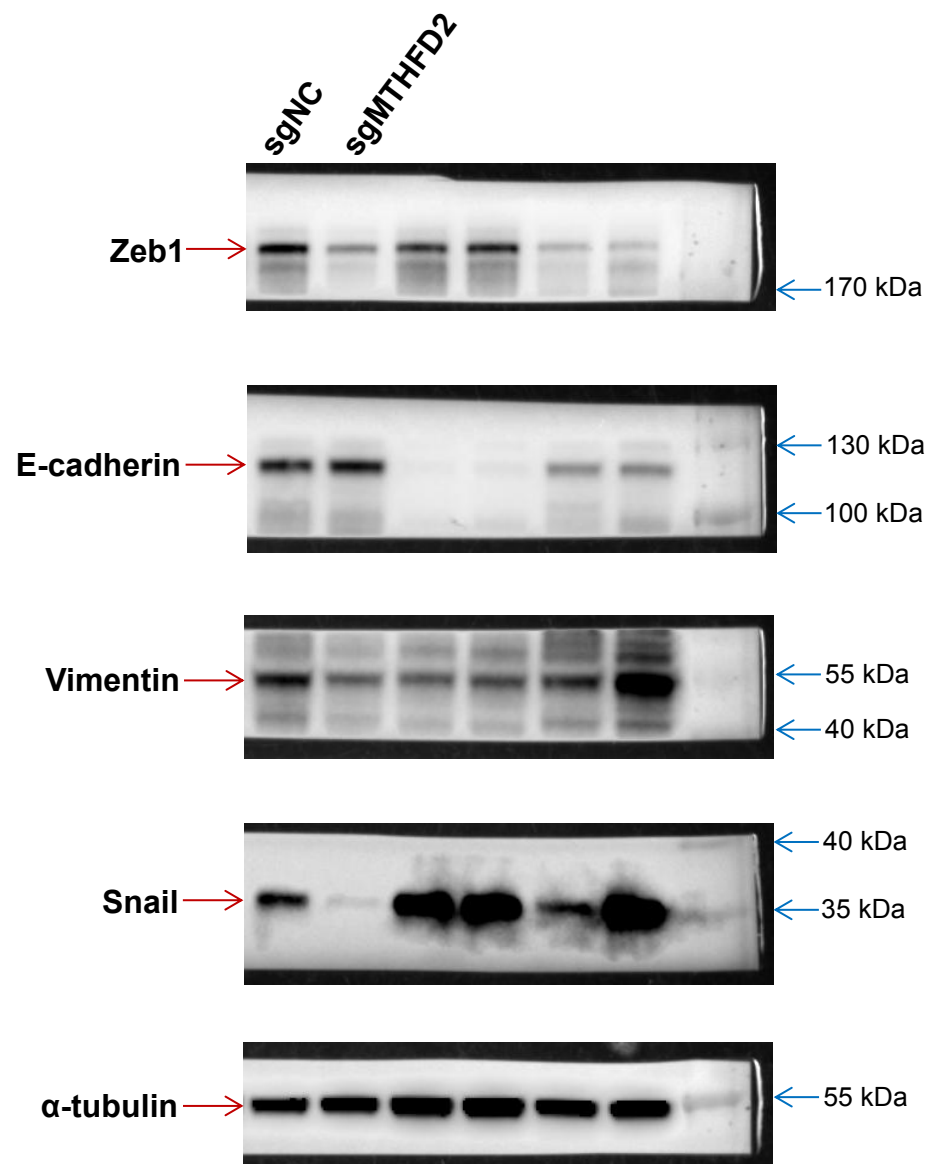

**Fig 6C**

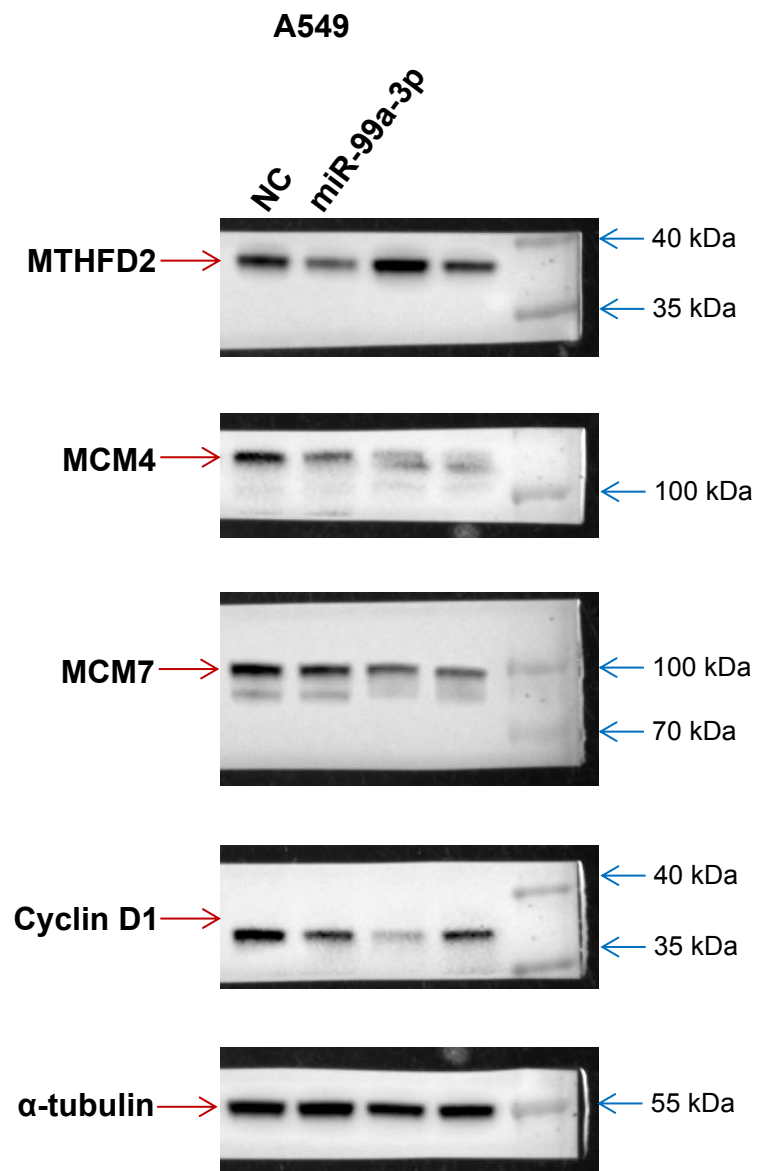

**Fig 6D**

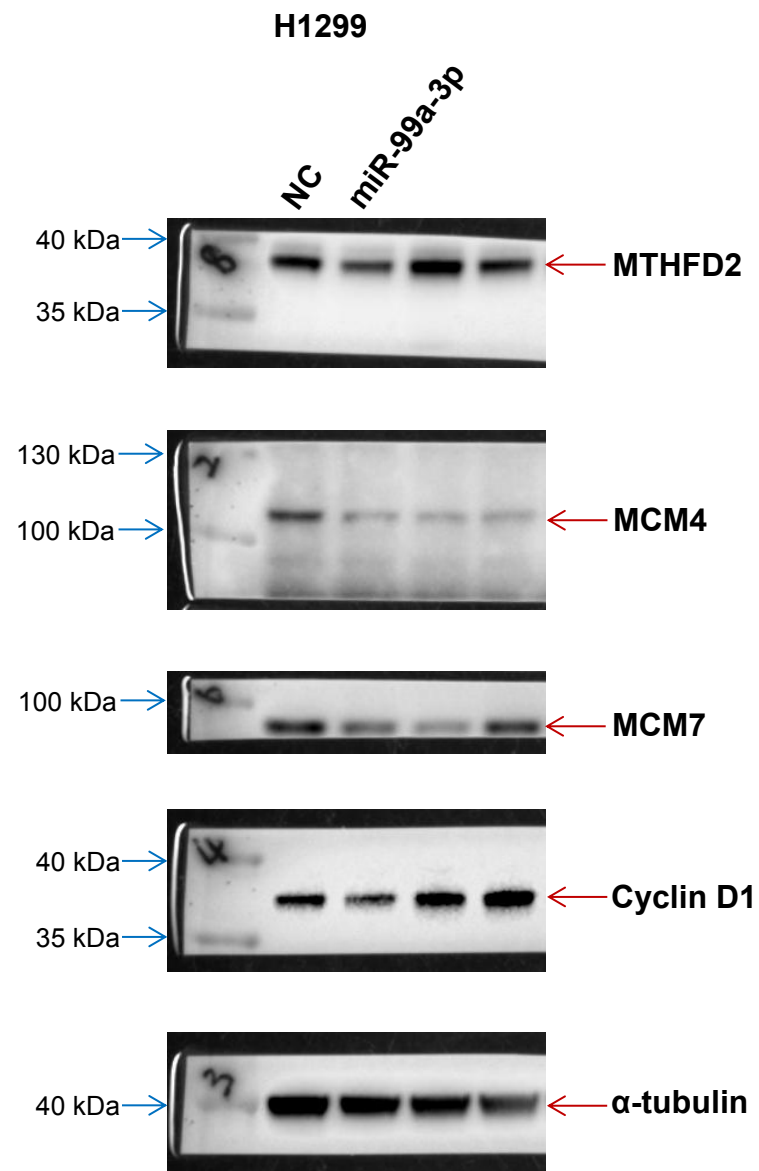

**Fig 6E**

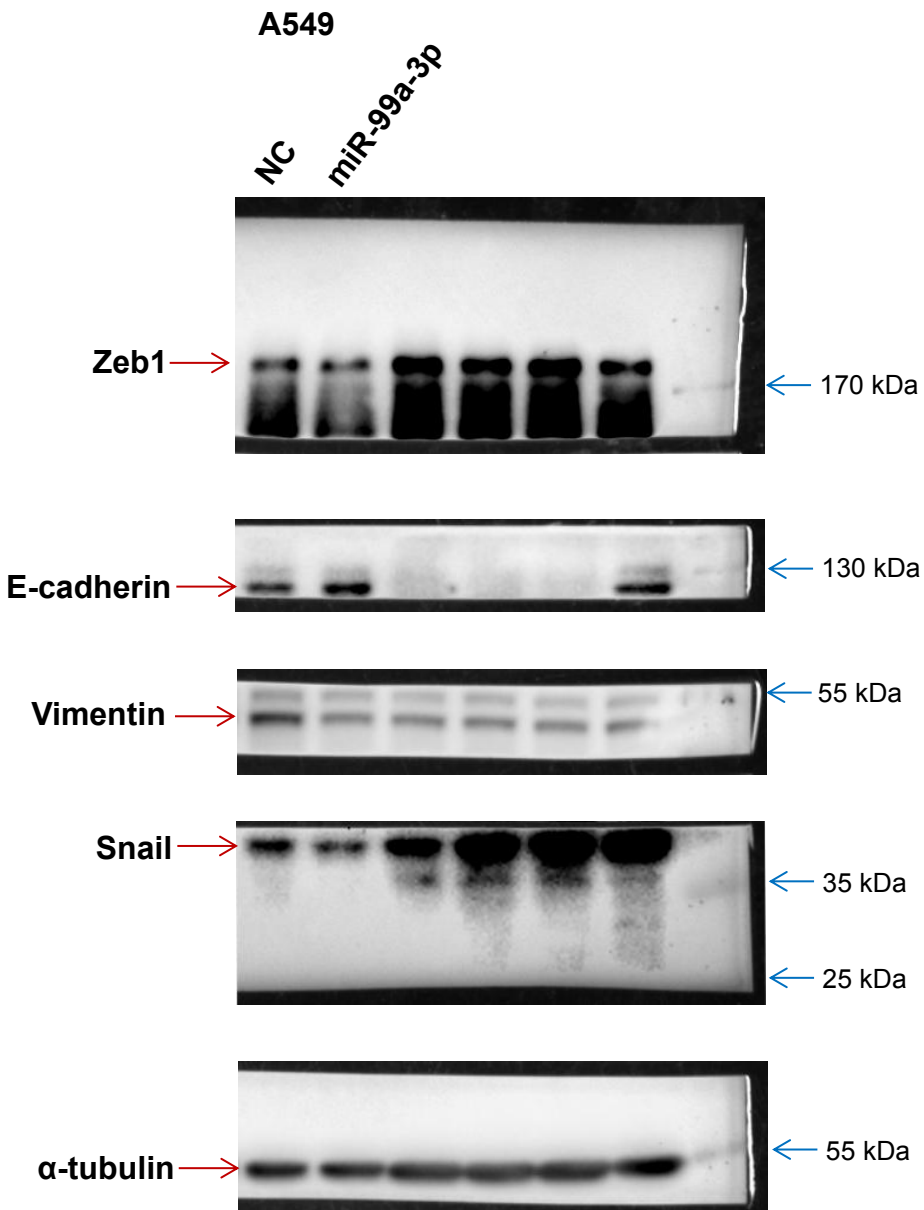

**Fig 6F**

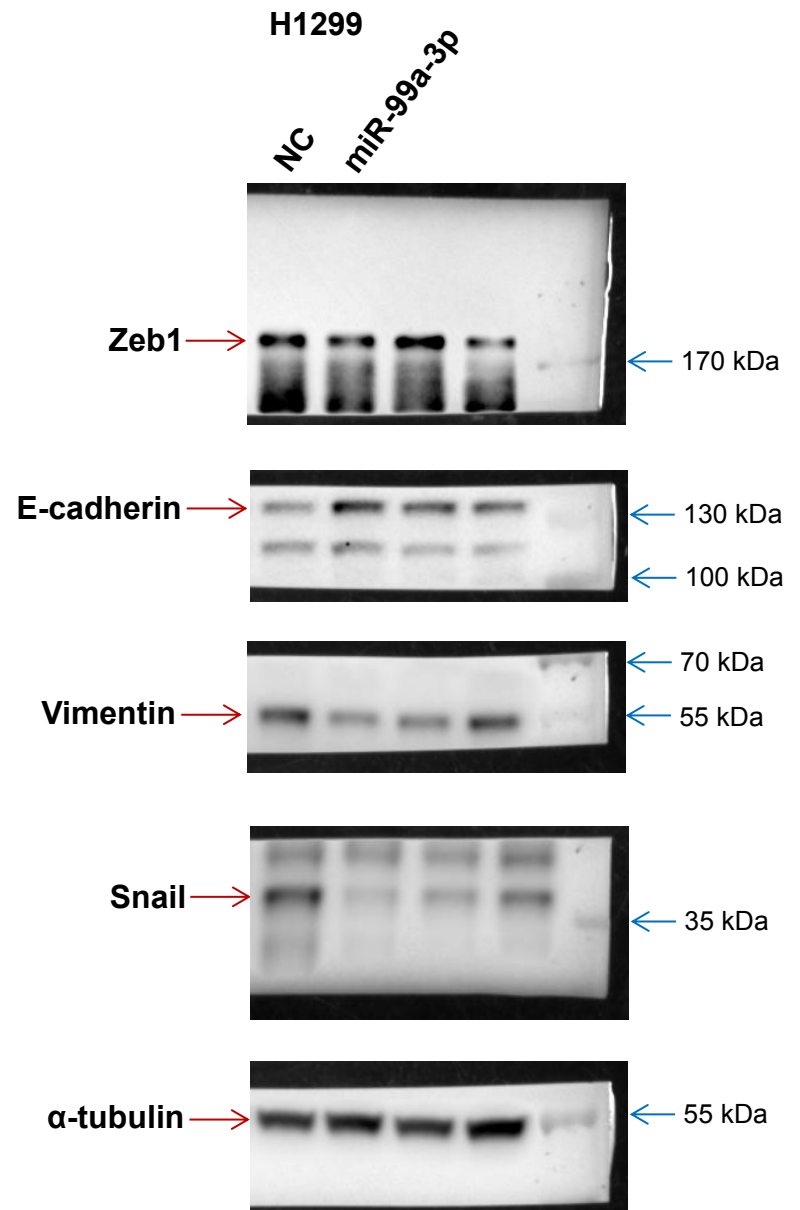

**Fig S1A**

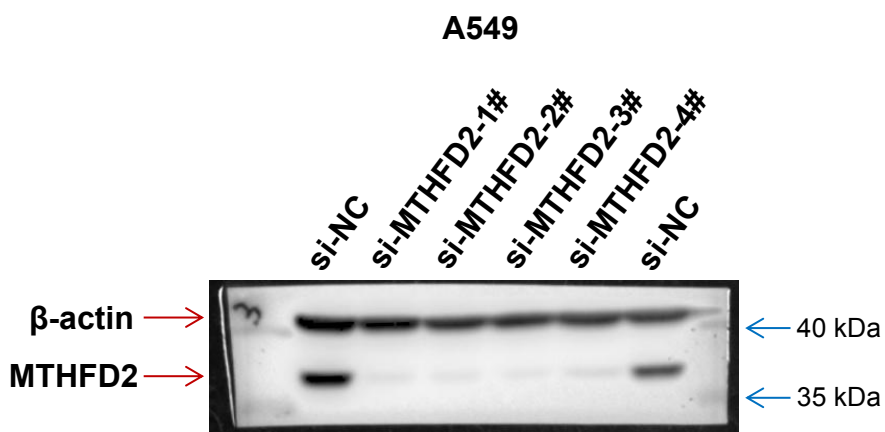

**Fig S1B**

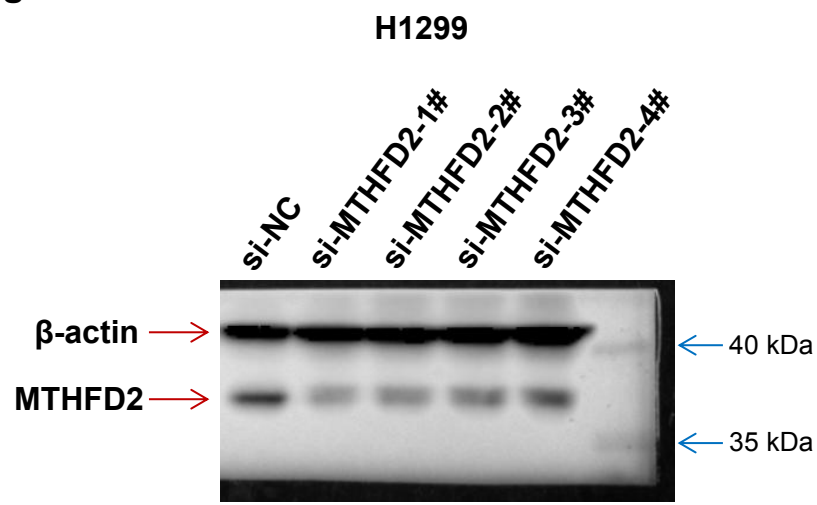

**Fig S2**

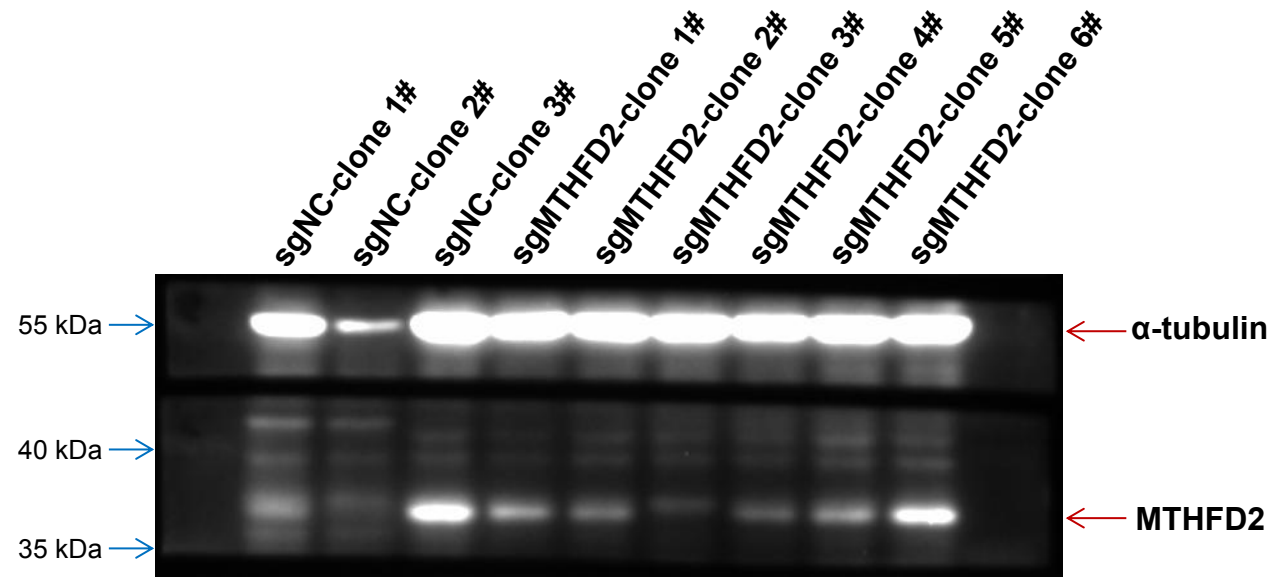

Supplement: Supplementary file 5 — Original western blots [file 41420_2022_1098_MOESM5_ESM.pdf]
