# Supplementary material for: Targeting mitochondrial one-carbon enzyme MTHFD2 together with pemetrexed confers therapeutic advantages in lung adenocarcinoma
Source: Cell Death Discov. 2022 Jul 5;8:307. doi: 10.1038/s41420-022-01098-y (PMC9256677; doi:10.1038/s41420-022-01098-y)
Supplement: Supplementary file 3 — Supplemental Tables [file 41420_2022_1098_MOESM3_ESM.docx]

**Supplementary Table 1. siRNA sequences targeting *MTHFD2*.**

| **Gene** | **Sequence** |
| --- | --- |
| *si-NC* sense | 5’-UUCUCCGAACGUGUCACGUTT-3’ |
| *si-NC* antisense | 5’-ACGUGACACGUUCGGAGAATT-3’ |
| *si-MTHFD-*1# sense | 5’-GCCUCUUCCAGAGCAUAUUTT-3’ |
| *si-MTHFD-*1# antisense | 5’-AAUAUGCUCUGGAAGAGGCTT-3’ |
| *si-MTHFD-*2# sense | 5’-GCGAGAAUCCUGCAAGUCATT-3’ |
| *si-MTHFD-*2# antisense | 5’-UGACUUGCAGGAUUCUCGCTT-3’ |
| *si-MTHFD-*3# sense | 5’-GAGCAGUUGAAGAAACAUATT-3’ |
| *si-MTHFD-*3# antisense | 5’-UAUGUUUCUUCAACUGCUCTT-3’ |
| *si-MTHFD-*4# sense | 5’-GGAUCAGUAUUCCAUGUUATT-3’ |
| *si-MTHFD-*4# antisense | 5’-UAACAUGGAAUACUGAUCCTT-3’ |

**Supplementary Table 2. sgRNA sequences against *MTHFD2*.**

| **Gene** | **Sequence** |
| --- | --- |
| *sgMTHFD-*1# sense | 5’-CACCGCGCCAACCAGGATCACACTC-3’ |
| *sgMTHFD-*1# antisense | 5’-CGCGGTTGGTCCTAGTGTGAGCAAA-3’ |
| *sgMTHFD-*2# sense | 5’-CACCGTGGGAAATAATCAAGCGAAC-3’ |
| *sgMTHFD-*2# antisense | 5’-CACCCTTTATTAGTTCGCTTGCAAA-3’ |
| *sgMTHFD-*3# sense | 5’-CACCGCGAAGGGAGCAGCTGTGCGC-3’ |
| *sgMTHFD-*3# antisense | 5’-CGCTTCCCTCGTCGACACGCGCAAA-3’ |

**Supplementary Table 3. Primer sequences for qRT-PCR.**

| **Gene** | **Forward Sequence** | **Reverse Sequence** |
| --- | --- | --- |
| *MTHFD2* | GATCCTGGTTGGCGAGAATCC | TCTGGAAGAGGCAACTGAACA |
| *MCM4* | AATCTTCTTTGACCGTTACCCTGAC | ATGAGCTGGTCAATGTCTTCTGGAT |
| *MCM7* | ACATCACAGCAGCATACG | TCCACCACATCCACCATT |
| *Cyclin D1* | GCTGCGAAGTGGAAACCATC | CCTCCTTCTGCACACATTTGAA |
| *ATP5G3* | TCAGACCAGTGCAATCAGCA | GCGAAGGGTTTCTGGCATAAC |
| *Vimentin* | GATGTTTCCAAGCCTGACCT | CACTTCACAGGTGAGGGACT |
| *ZEB1* | GCACAACCAAGTGCAGAAGA | CATTTGCAGATTGAGGCTGA |
| *SNAI1* | AATCGGAAGCCTAACTACAGCGAG | CTTTCCCACTGTCCTCATCTGACA |
| *miR-99a-3p* | GATGCAAGCTCGCTTCTATG | CAGTGCGTGTCGTCCAGT |
| *U6* | GGAACGATACAGAGAAGATTAGC | TGGAACGCTTCACGAATTTGCG |
| *β-actin* | GGATGCAGAAGGAGATCACTG | CGATCCACACGGAGTACTTG |

**Supplementary Table 4. Detailed information of the antibodies.**

| **Name** | **Host species** | **Source** | **Identifier** | **dilution** | **Molecular weight** | **Applications** |
| --- | --- | --- | --- | --- | --- | --- |
| Zeb1 | Rabbit | Cell Signaling Technology | #70512 | 1:500 | 200 KD | WB |
| E-cadherin | Rabbit | Cell Signaling Technology | #3195 | 1:500 | 135KD | WB |
| Vimentin | Rabbit | Cell Signaling Technology | #5741 | 1:500 | 57KD | WB |
| Snail | Rabbit | Cell Signaling Technology | #3879 | 1:500 | 35KD | WB |
| Cyclin D1 | Rabbit | Cell Signaling Technology | #2978 | 1:500 | 36KD | WB |
| α-tubulin | Mouse | Cell Signaling Technology | #3873 | 1:2000 | 54KD | WB |
| MTHFD2 | Mouse | Santa Cruz | sc-100750 | 1:200 | 38KD | WB, IHC |
| MCM4 | Mouse | Santa Cruz | sc-28317 | 1:100 | 100KD | WB |
| MCM7 | Mouse | Santa Cruz | sc-9966 | 1:200 | 88KD | WB |
| β-actin | Mouse | EarthOX | E-021020-01 | 1:2000 | 43KD | WB |
