# Supplementary material for: Targeting mitochondrial one-carbon enzyme MTHFD2 together with pemetrexed confers therapeutic advantages in lung adenocarcinoma
Source: Cell Death Discov. 2022 Jul 5;8:307. doi: 10.1038/s41420-022-01098-y (PMC9256677; doi:10.1038/s41420-022-01098-y)
Supplement: Supplementary file 4 — Supplemental Figure and Table legends [file 41420_2022_1098_MOESM4_ESM.docx]

**Supplementary Figure and Table legends**

**Fig. S1 Verification of knockdown efficiencies of MTHFD2 from different siRNAs. A, B** A549 and H1299 cells were transfected with siRNA scramble and four different MTHFD2 siRNAs for 72 h. Western blot analysis was conducted to validate the knockdown efficiencies of MTHFD2 in A549 and H1299 cells. The quantification of MTHFD2 band density relative to β-actin was shown below. ***P* < 0.01, ****P* < 0.001 vs. si-NC group.

**Fig. S2 Selection of successful MTHFD2-knockout clone by CRISPR/Cas9 system.** A549 cells were transfected with virus derived from 293T cell supernatant containing sgRNA scramble and three different MTHFD2 sgRNAs. Stable express cells were obtained by adding 400 ng/mL puromycin. Cells were sorted and transferred to ninety-six well plates at the density of one single cell per well. Western blot analysis was performed to select the successful MTHFD2-knockout cell clone. The quantification of MTHFD2 band density relative to α-tubulin was shown below. **P* < 0.05, ***P* < 0.01, ****P* < 0.001 vs. sgNC clone 1 group.

**Table S1. siRNA sequences against *MTHFD2*.**

**Table S2. sgRNA sequences targeting *MTHFD2*.**

**Table S3. Primer sequences for** **qRT-PCR.**

**Table S4. Detailed information of the antibodies.**
